# From Ethnobotany to Pharmacology: A Comprehensive Review of Selected Asteraceae Species Highlighting Phytochemical Composition, Traditional Uses, and Diverse Pharmacological Activities

**DOI:** 10.3390/ph19081220

**Published:** 2026-08-03

**Authors:** Joy Khoury, Magda Lena El Dada, Heba Hellany, Ghosoon Albahri, Rola Abdallah, Raya I. Hage, Mikhael Bechelany, Elias Baydoun

**Affiliations:** 1 Department of Biology, Faculty of Arts and Sciences, American University of Beirut, Riad El Solh, Beirut 1107 2020, Lebanon; jsk19@mail.aub.edu (J.K.); mle02@mail.aub.edu (M.L.E.D.); he115@aub.edu.lb (H.H.); ga159@aub.edu.lb (G.A.); rha62@mail.aub.edu (R.A.); rih30@mail.aub.edu (R.I.H.); 2 Institut Européen des Membranes (IEM), UMR-5635, CNRS, ENSCM, Université de Montpellier, Place Eugene Bataillon, 34095 Montpellier, France; 3 Gulf Department of Mathematics and Natural Sciences, Gulf University for Science and Technology (GUST), Hawally 32093, Kuwait; 4 Marine Science Station, University of Jordan, Amman 11942, Jordan

**Keywords:** Asteraceae family, traditional medicine, phytochemicals, anti-cancer, anti-inflammatory, green nanotechnology

## Abstract

Medicinal plants are widely used in the Mediterranean region, where they have long been employed in traditional medicine as well as in the food and beverage industry. At the same time, people in developed countries are increasingly dissatisfied with modern healthcare due to the side effects and complications associated with chemical drugs, prompting a growing interest in more natural alternative treatments. The Asteraceae family is one of the largest flowering plant families, containing many species with medicinal properties. It comprises over 1600 genera and approximately 25,000 species worldwide. *Silybum marianum*, *Picnomon acarna*, *Glebionis segetum*, and *Carduus pycnocephalus* are invasive species in the Mediterranean region belonging to the Asteraceae family. Although they have been used traditionally to treat certain diseases, their biological and pharmacological potential has not yet been fully explored. This review aims to provide a comprehensive overview of the phytochemical components, geographical and botanical characteristics, traditional uses, pharmacological activities, and potential applications of these species in green nanotechnology and the food industry.

## 1. Introduction

Invasive plant species represent a major ecological and agricultural concern worldwide, particularly in Mediterranean and semi-arid ecosystems where climatic conditions favor their establishment and spread [1]. Several members of the Asteraceae family have attracted considerable attention due to their adaptability, persistence, and influence on agroecosystems [2]. The Asteraceae family is one of the largest and most diverse families of flowering plants, comprising an estimated 25,000–30,000 species distributed across a wide range of ecological habitats [3]. Members of this family are particularly well represented among agricultural weeds because of their prolific seed production, efficient dispersal mechanisms, and remarkable adaptability to disturbed environments [4]. Beyond their ecological success, Asteraceae species are recognized as a rich source of structurally diverse bioactive secondary metabolites, including sesquiterpene lactones, flavonoids, phenolic acids, and terpenoids, which have been associated with a broad range of pharmacological activities, such as anti-oxidant, anti-inflammatory, anti-microbial, and anti-cancer effects [3,5,6]. This unique combination of ecological abundance and phytochemical richness positions the Asteraceae family as an attractive target for natural product research, transforming plants often regarded as agricultural weeds into valuable sources of therapeutic compounds [6,7]. However, despite increasing interest in the medicinal potential of individual species, comparative studies evaluating the phytochemical diversity and pharmacological properties of co-occurring weedy Asteraceae species remain limited, underscoring the need for the present synthesis. Among these, *Picnomon acarna* is widely distributed across Mediterranean countries and is frequently encountered in cultivated and managed landscapes, where it contributes to shaping plant community dynamics and agricultural practices [8]. Similarly, *Silybum marianum* has gained recognition not only for its long-standing medicinal value but also for its broad geographic distribution beyond its native range. Its widespread introduction across continents reflects both its biological resilience and its importance as a plant of economic and pharmacological interest [9]. Another notable species, *Carduus pycnocephalus*, has similarly expanded beyond its Mediterranean origin and is now commonly observed in diverse regions worldwide, contributing to vegetation diversity in disturbed and managed environments [10]. In contrast, *Glebionis segetum* represents a species whose presence has declined in certain parts of its native range, despite its historical association with agricultural systems [11]. Numerous modern pharmaceuticals are derived directly or indirectly from plant secondary metabolites, highlighting the importance of botanical resources in drug discovery and development. The continued exploration of plant-based compounds offers significant potential for identifying novel anti-microbial, anti-oxidant, and anti-inflammatory agents [12]. Moreover, the growing concern over anti-biotic resistance has renewed interest in medicinal plants as alternative or complementary therapeutic options [13]. Therefore, investigating the phytochemical and pharmacological components of plant species, including those traditionally regarded as weeds, represents an important approach in expanding the repertoire of natural bioactive compounds and supporting the development of safer and more sustainable treatments.

## 2. Taxonomical Classification (Index Kewensis, POWO)

The taxonomic classification of the plants was obtained from the Plants of the World Online (POWO) website and the Index Kewensis.

a *Picnomon acarna*

Kingdom: Plantae; Phylum: Streptophyta; Class: Equisetopsida; Order: Asterales; Family: Asteraceae; Genus: Picnomon; Species: *Picnomon acarna* (L.) Cass.

b *Silybum marianum*

Kingdom: Plantae; Phylum: Streptophyta; Class: Equisetopsida; Order: Asterales; Family: Asteraceae; Genus: Silybum; Species: *Silybum marianum* (L.) Gaertn.

c *Carduus pycnocephalus*

Kingdom: Plantae; Phylum: Streptophyta; Class: Equisetopsida; Order: Asterales; Family: Asteraceae; Genus: Carduus; Species: *Carduus pycnocephalus* Spreng.

d *Glebionis segetum*

Kingdom: Plantae; Phylum: Streptophyta; Class: Equisetopsida; Order: Asterales; Family: Asteraceae; Genus: Glebionis; Species: *Glebionis segetum* (L.) Fourr.

## 3. Methods

A comprehensive literature search was conducted using major scientific databases, including PubMed, Scopus, ScienceDirect, SciFinder, Chemical Abstracts, Medicinal and Aromatic Plants Abstracts, to identify relevant articles on the review topic. Additional sources were retrieved through general web searches using Google and Google Scholar (accessed using Google Chrome, version 138.0). The search included publications from 1988 to the end of 2025 and was performed using relevant keywords and MeSH terms related to ‘*Picnomon acarna*’, ‘*Silybum marianum*’, ‘*Carduus pycnocephalus*’, ‘*Glebionis segetum*’, AND (‘traditional uses’, ‘ phytochemical components’, ‘pharmacological applications, or roles, or effects’, ‘antimicrobial’, ‘anticancer’, ‘antioxidant’, ‘antidiabetic’, ‘anti-enzymatic’, and ‘anti-obesity’, ‘anti-inflammatory’, ‘neuroprotective’, ‘green nanotechnology’, ‘food’, ‘toxicity’).

The inclusion criteria were the studies investigating the extracts of the four selected species as the intervention, with outcomes focusing on their pharmacological applications, potential nanotechnology applications, and food-related applications. Studies involving all experimental approaches, including in vitro, in vivo, and in silico investigations, were considered eligible. The exclusion criteria included studies focusing on plant species other than the selected species, non-English publications, and studies investigating isolated compounds derived from the selected species rather than the complete plant extracts. Differences were observed in the plant parts used (e.g., leaves, flowers, seeds, and aerial parts), extraction methods and solvents, extract concentrations, experimental conditions, biological models, outcome measures, and analytical techniques.

The limitations of the experimental models used in the included studies were also considered during data interpretation. Most pharmacological evidence was derived from in vitro assays or animal models, which may not fully reflect human physiological responses.

**Figure 1:**
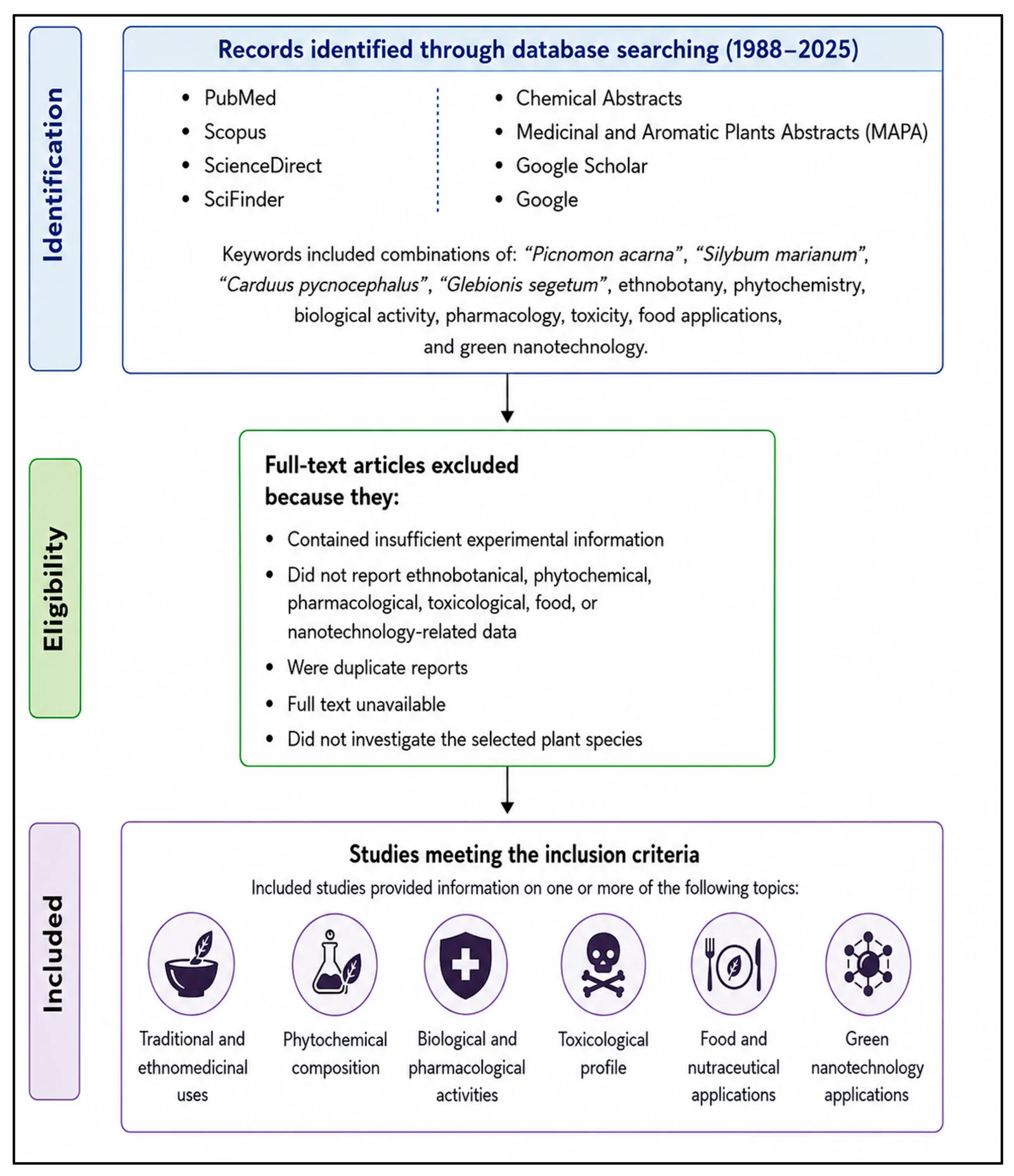
Flow diagram illustrating the literature search and study selection process used in this comprehensive review. Articles published between 1988 and 2025 were identified through multiple scientific databases. After duplicate removal, studies were screened based on predefined inclusion and exclusion criteria, followed by a full-text eligibility assessment. Eligible publications reporting ethnobotanical, phytochemical, pharmacological, toxicological, food, or green nanotechnology applications of *Picnomon acarna*, *Silybum marianum*, *Carduus pycnocephalus*, and *Glebionis segetum* were included in the review.

Duplicate records were removed manually, and the eligible studies were used for data extraction and analysis. A flow diagram illustrating the literature search strategy has been added (Figure 1).

## 4. Geographical and Botanical Characteristics

### 4.1. Geographical and Ecological Characteristics

*Picnomon acarna* is an invasive thistle species native to the Mediterranean region, where it is widely distributed and recognized as an aggressive agricultural weed [14,15]. Its natural range extends across numerous countries, including Lebanon, where it is locally known as “shawk-ul-far” and classified as a principal weed along with Syria, Turkey, Iran, Iraq, Albania, Bulgaria, Crete, France, Greece, Italy, Portugal, Romania, Russia (Crimea), Sardinia, Switzerland, Spain, and the former Yugoslavia. Outside its native range, *P. acarna* has expanded into regions such as Morocco and Australia, where it is known as soldier thistle and poses serious challenges in agricultural landscapes, particularly in the Victorian Mallee and Wimmera regions [14]. Ecologically, *P. acarna* is an annual weed well adapted to rainfed agricultural systems and disturbed habitats, including crop fields, gardens, roadsides, riverbanks, and abandoned land [16]. It thrives in areas receiving annual rainfall between 300 and 600 mm and exhibits low palatability, high tolerance to grazing pressure, and resistance to soil compaction. These characteristics have contributed to increased plant density in rangelands and forest ecosystems of western Iran. The species negatively affects agricultural productivity by reducing yields of crops such as wheat and chickpea, while its spiny morphology significantly interferes with harvesting operations, occasionally leading farmers to abandon chickpea and lentil fields [17]. Reproduction occurs exclusively through seeds. The seeds are efficiently dispersed by wind via a pappus measuring 1–2 cm in length and are also spread unintentionally by animals, vehicles, irrigation water, mud, wool, and hides. Additionally, the entire plant may detach and act as a tumbleweed, enhancing seed dispersal [17]. Freshly produced seeds exhibit dormancy and require a post-ripening period of approximately 12 weeks under dry conditions before achieving maximum germination. Germination is strongly light-dependent due to positive photoblastism and occurs over a broad temperature range (5–35 °C), with optimal germination (up to 95%) observed under alternating temperatures of 20/10 °C [14]. The species tolerates wide pH conditions (4–10), preferring alkaline soils, and shows high resistance to water stress, making it well suited to drought-prone rainfed systems. However, it is sensitive to salinity, with 50% germination inhibition recorded at 20.53 mM NaCl. Seedling emergence is restricted by burial depth, with no emergence occurring beyond 4 cm, indicating that minimum tillage favors its spread, whereas deep tillage may effectively suppress its establishment [14]. *Silybum marianum* (milk thistle) originates from the Mediterranean regions of Europe and Asia [18]. Due to its long-standing medicinal use and subsequent cultivation, it has been widely introduced beyond its native range and is now considered invasive in many regions worldwide, including parts of North and South America, southern Australia, and various areas across Europe and Asia [19]. In Canada, the species is present in several provinces, including British Columbia, Alberta, Manitoba, Ontario, Quebec, New Brunswick, and Nova Scotia, although its distribution in British Columbia is largely restricted to the southwest coast. It is classified as a noxious weed under the British Columbia Weed Control Act [20]. Milk thistle is a highly adaptable annual species with strong reproductive capacity and shade tolerance, allowing it to colonize a wide range of disturbed environments [20]. It commonly forms dense populations in abandoned lands, roadsides, pastures, and nutrient-rich soils such as meadows. Its invasive growth habit enables it to outcompete native vegetation and desirable forage species, thereby reducing grazing capacity. The plant’s sharp spines pose risks to livestock and humans and provide shelter for agricultural pests. Furthermore, nitrate accumulation within plant tissues may cause toxicity in grazing animals [21]. *S. marianum* exhibits considerable phenotypic plasticity, allowing it to adapt to variations in climate, altitude, and soil conditions [22]. Elevation plays a key role in determining habitat suitability, with lower altitudes generally supporting higher population densities. In Iran, particularly in Fars Province, lower elevations in the northwest, west, and south have been identified as highly suitable for growth [22]. Climatic factors such as temperature and rainfall significantly influence both plant distribution and silymarin accumulation. While moderate temperatures and rainfall favor plant growth, higher temperatures combined with reduced precipitation enhance silymarin production [18]. Soil parameters also influence plant performance, with higher importance values associated with alkaline soils rich in organic matter and nitrogen, particularly at lower elevations [22]. At higher altitudes, the plant allocates more resources to reproduction rather than vegetative growth, ensuring reproductive success under less favorable conditions [20]. Reproduction occurs solely via seeds. Each flower head can produce approximately 100 seeds, resulting in 1000–5000 seeds per plant. The seeds are dark brown, oval achenes with a golden ring and remain viable in the soil seed bank for over nine years, contributing substantially to the species’ invasiveness [23]. Although seeds possess a pappus that facilitates wind dispersal, their relatively heavy mass limits long-distance movement. Human-mediated dispersal via roads, vehicles, water, animals, and agricultural equipment plays a major role in seed spread, with higher population densities frequently observed near road networks [22]. *Carduus pycnocephalus* (Italian thistle) is an annual or biennial herb native to the Mediterranean region, including southern Europe, northern Africa, and western Asia [24]. It has since been widely introduced and is now considered invasive in regions such as California, Australia, and South America. The species thrives in disturbed habitats, including pastures, croplands, roadsides, wastelands, and riparian zones. In California, it is commonly found in areas receiving 250–1000 mm of annual rainfall and at elevations below 1500 m. Due to its competitive growth and dense spiny structure, *C. pycnocephalus* significantly reduces pasture productivity and deters grazing animals. Reproduction occurs exclusively by seeds, which are dispersed through wind, animals, vehicles, and contaminated agricultural materials. Seeds generally remain viable in the soil for several years [15,25]. *Glebionis segetum* (corn marigold) is predominantly distributed along the western coastal regions of Great Britain, particularly in areas characterized by well-drained sandy and shale-based soils, including Cornwall, Devon, Pembrokeshire, Ceredigion, Gower, and the Outer Hebrides [26]. It is commonly associated with cultivated agricultural land but is also found in disturbed habitats such as roadsides, wastelands, refuse sites, and overgrazed fields [26]. Ecologically, *G. segetum* is an annual species that flowers between June and October. It shows a preference for light, well-drained, slightly acidic soils but can also tolerate calcareous substrates and a range of nutrient conditions [27]. Seed germination is closely linked to agricultural disturbance, particularly soil cultivation, which brings dormant seeds to the surface. Seedlings may emerge in early spring, while those emerging in autumn can survive frost conditions. The species plays an important ecological role by supporting pollinators such as bees, butterflies, and hoverflies [28]. Due to a marked population decline, *G. segetum* is currently classified as vulnerable in Great Britain and England. This decline is largely attributed to intensified agricultural practices, including herbicide application, seed cleaning, liming, and changes from spring to autumn cropping systems. Conservation efforts emphasize the need for annual soil disturbance and shallow seed placement to promote germination and sustain remaining populations [29].

### 4.2. Botanical Characteristics: Roots, Leaves, and Stem

**Figure 2:**
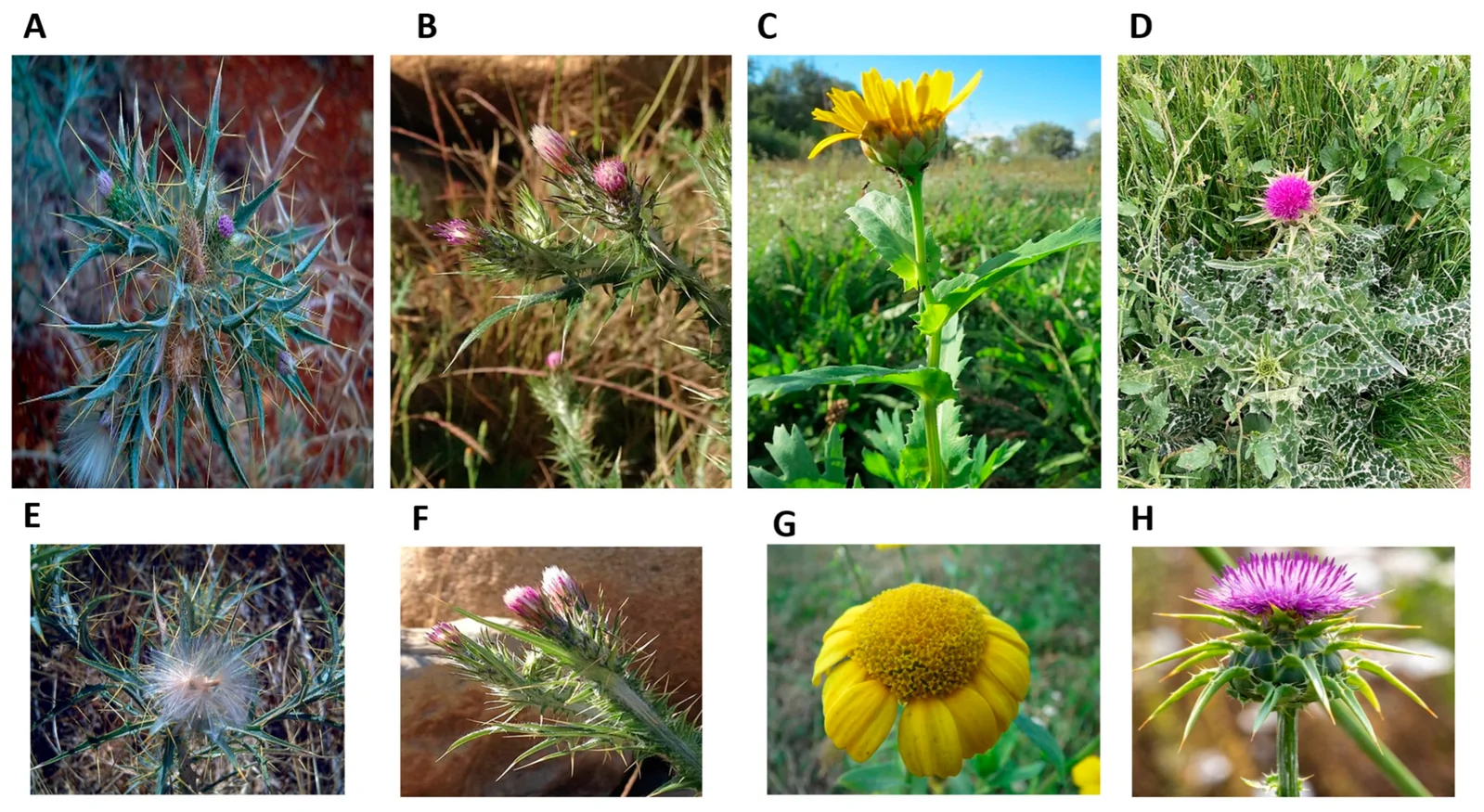
(**A**,**E**) *Picnomon acarna*, (**B**,**F**) *Carduus pycnocephalus*, (**C**,**G**) *Glebionis segetum*, (**D**,**H**) *Silybum marianum* (**A**,**E**) reproduced from https://commons.wikimedia.org/wiki/User:Javier_martin (accessed on 26 July 2026) (2008), licensed under https://en.wikipedia.org/wiki/en:public_domain (accessed on 26 July 2026), (**B**,**F**) reproduced from https://commons.wikimedia.org/wiki/File:Italian_Thistle.jpg?wprov=srpw1_0 (accessed on 26 July 2026) (2016), licensed under https://creativecommons.org/publicdomain/zero/1.0/deed.en (accessed on 26 July 2026), (**C**,**G**) reproduced from https://commons.wikimedia.org/wiki/User:AnRo0002 (accessed on 26 July 2026) (2013), licensed under https://creativecommons.org/publicdomain/zero/1.0/deed.en (accessed on 26 July 2026), (**D**,**H**) reproduced from https://commons.wikimedia.org/wiki/User:Awinch1001 (accessed on 26 July 2026) (2026), licensed under https://creativecommons.org/publicdomain/zero/1.0/deed.en (accessed on 26 July 2026).

*Picnomon acarna* (L.) Cass., commonly known as soldier thistle, is an erect annual member of the Asteraceae that can reach up to 100 cm in height and is distinguished by its densely branched, winged, and strongly spiny stems covered with thick, cobweb-like hairs that give the plant a wooly appearance [30]. The leaves are densely pubescent with white fibers; basal rosette leaves may reach 30 cm in length and are weakly lobed with short spines, while the smaller cauline leaves (up to 10 cm) are narrow, oppositely arranged, and bear prominent yellow spines measuring 10–15 mm along the lobes, with additional smaller spines along the margins. Leaf bases extend downward along the stem, forming spiny wings that enhance protection and structural rigidity [17]. *Silybum marianum* (milk thistle) is characterized by a strong, deep taproot with central yellow wood and well-developed latex vessels within a thick bark, enabling persistence in nutrient-rich and disturbed soils typical of Mediterranean habitats [31]. The plant develops large, glossy, dark green leaves with conspicuous milky-white venation; basal rosette leaves are obovate and deeply lobed, reaching up to 50 cm, while alternately arranged cauline leaves are sessile, lanceolate to oblong, and armed with rigid spines along pinnatifid margins [31]. The erect stem is grooved, hollow, smooth to slightly pubescent, unwinged, and often striped, reaching 30–200 cm in height, branching in the upper portions, and exuding milky latex upon injury [25]. *Carduus pycnocephalus* (Italian thistle) possesses a sturdy taproot with fibrous lateral roots, which provide stability and facilitate rapid establishment in disturbed Mediterranean grasslands and open woodlands. Its leaves are lanceolate to oblong (10–25 cm long), deeply pinnatifid with a leathery texture and sharp yellow spines along the margins, and are directly attached to the stem, with a cottony tomentum on the abaxial surface [32]. The upright stem, reaching 60–150 cm, is ridged, densely pubescent, and winged due to decurrent leaf bases; it is typically unbranched at the base and branched toward the apex, contributing to its competitive growth habit in invaded habitats [33]. *Glebionis segetum* (corn marigold) exhibits a fibrous root system well adapted to sandy, cultivated, and disturbed soils common in coastal and temperate regions of Europe [34]. Its leaves are alternately arranged, oblanceolate to spatulate (3–10 cm long), with irregularly toothed or pinnatifid margins and smooth to slightly hairy surfaces. The stems are erect, freely branching, and reach heights of 15–60 cm; they are silky to weakly glandular, distinctly striated, and often display reddish pigmentation, facilitating field identification and reflecting adaptation to open agricultural environments [35]. Figure 2 shows the morphological diversity of the four species, highlighting the variations in their flowers.

## 5. Traditional Uses of Each Plant

*Picnomon acarna* (L.) Cass., the sole species of its genus within the Asteraceae family, has a long history of traditional use across the Mediterranean basin and the Middle East, particularly for gastrointestinal, musculoskeletal, and inflammatory disorders. In Lebanon, rural communities around Mount Hermon prepare decoctions of the aerial parts to treat gastric pain and rheumatism, with high informant consensus reflecting strong ethnomedicinal reliability, while in Iran, Kurdish populations use the leaves internally as a stomachic for indigestion and gastric discomfort; additional ethnobotanical records from Turkey report its use as an infusion for cancer treatment, and Greek folk medicine describes the plant as a stimulant, tonic, and antiseptic, uses that are partially supported by the identification of bioactive flavonoid methyl ethers in its leaves and flowers [36,37]. *Silybum marianum* (L.) Gaertn. (milk thistle), another Asteraceae species native to the Mediterranean region, possesses one of the most extensively documented traditions of medicinal use, dating back more than 2000 years, primarily targeting liver and biliary disorders; historical sources from classical antiquity through the Middle Ages describe its application for bile flow, liver detoxification, and poisoning, while modern traditional medicine systems employ its fruits and seeds—rich in silymarin—for hepatitis, cirrhosis, jaundice, and toxin-induced liver injury, with additional uses as a galactagogue, digestive aid, diuretic, antidote to mushroom poisoning, and remedy for dermatological and gynecological conditions across Europe, Iran, and, later, North America [23,25]. *Carduus pycnocephalus* (L.) Gaertn., an annual or biennial thistle native to the Mediterranean, is traditionally valued both as food and medicine; ethnobotanical data from Iran and other Mediterranean and Asian regions describe its use as a liver and spleen tonic, digestive, diuretic, and remedy for constipation, headache, stomachache, diarrhea, and respiratory complaints, while broader traditional knowledge attributes anti-inflammatory, antispasmodic, and anti-microbial properties to the genus *Carduus*, uses supported by modern pharmacological observations [33,35,36,37,38,39,40,41]. *Glebionis segetum* (L.) Fourr. (syn. *Chrysanthemum segetum* L.) is widely distributed across Europe, North Africa, Asia, and the Mediterranean and has been traditionally employed both as a medicinal and edible plant; in Anatolia, it is used to alleviate abdominal pain, sore throat, shortness of breath, and hair loss, while its aerial parts are consumed as salad, and traditional Asian uses further include applications related to feeding deterrence and pest control, highlighting its multifaceted ethnobotanical relevance [26].

## 6. Phytochemical Components in the Four Plants

The identification of isolated compounds was carried out using various analytical techniques, including chromatographic methods such as TLC and LC-MS/MS, as well as spectroscopic techniques like UV–Vis and NMR. The first compounds identified in the Asteraceae family were flavonoids. The earliest study on the identification of flavonoids from the Asteraceae family was conducted on the aerial parts of *Picnomon acarna*, where three flavonoids were isolated via chromatographic separation, and their structures subsequently elucidated using chemical and spectroscopic methods [41]. Another study focused on the identification of flavonoids from *Silybum marianum*, where seven flavonoids were extracted from its flowers and identified using spectroscopic techniques [42].

The type of extraction method employed and the plant part analyzed can influence the phytochemicals identified. For instance, in a study investigating the chemical composition of *Silybum marianum* using an ethanol/water extract, 33 phytochemicals were identified, including flavonolignans (such as silybin, isosilybin, and silychristin), as well as phenolics, flavonoids, and tannins [43]. In contrast, another study examining the oil of the same plant reported the presence of unsaturated fatty acids and phytosterols [42]. Table 1 summarizes the major constituents identified from the four Asteraceae plants using various extraction solvents, demonstrating their significance as valuable sources of bioactive compounds.

## 7. Pharmacological Activities of Each Plant

### 7.1. Anti-Microbial Activity

#### 7.1.1. Anti-Microbial Activity of *Silybum marianum*

*Silybum marianum* extracts exhibited variable anti-microbial activity depending on the solvent, plant part, and extraction method [46]. Ethanolic seed extracts showed the highest anti-bacterial activity, particularly against MRSA and *Stenotrophomonas maltophilia*, while acetone extracts displayed moderate activity but were ineffective against Gram-negative bacteria [47]. Aqueous extracts, especially hot water, demonstrated weak or no activity, suggesting possible degradation or poor extraction of bioactive compounds [48]. In contrast, chloroform and n-butanol extracts exhibited bacteriostatic effects with moderate anti-bacterial and anti-fungal activities, with n-butanol showing greater potency based on lower MIC values. Hydroalcoholic extracts obtained via ultrasound-assisted extraction showed moderate, dose-dependent activity against both bacterial and fungal strains [48]. Methanolic extracts revealed that stems were generally more active than leaves and flowers, particularly against *Pseudomonas aeruginosa*. Similarly, acetone and ethyl acetate extracts showed variable but notable activity depending on the plant part, with flowers often being more effective. Petroleum ether extracts exhibited moderate anti-microbial effects but were less potent overall [49]. Collectively, these findings indicate that organic solvents, particularly ethanol and methanol, are more efficient in extracting anti-microbial compounds than aqueous solvents as shown in Table 2.

#### 7.1.2. Anti-Microbial Activity of *Carduus pycnocephalus*

*Carduus pycnocephalus* methanolic extracts demonstrated notable anti-microbial activity that varied by plant part, with leaves and flowers generally showing stronger inhibition compared to roots and stems, particularly against *Salmonella typhimurium* and *Bacillus cereus* [52]. Root and stem extracts exhibited moderate activity, especially against *Pseudomonas aeruginosa*, while showing no effect on certain strains such as *Staphylococcus haemolyticus* and *S. xylosus* [52]. Ethanolic extracts of aerial parts displayed broad-spectrum anti-microbial activity against both Gram-positive and Gram-negative bacteria and fungi, although no effect was observed against *Candida albicans*. In contrast, the essential oil showed selective activity with low MIC values against Gram-positive bacteria but no inhibitory effect against Gram-negative bacteria or *C. albicans*, highlighting the influence of extraction type on anti-microbial efficacy as shown in Table 3.

### 7.2. Anti-Oxidant Activity

Oxidative stress arises from an imbalance between the production of reactive oxygen species (ROS) and the body’s endogenous anti-oxidant defenses. While ROS are naturally generated under normal physiological conditions, their accumulation can trigger oxidation, leading to cellular damage and various diseases [53]. During oxidative stress, anti-oxidant enzymes—including superoxide dismutase (SOD), catalase (CAT), glutathione peroxidase (GPx), and glutathione reductase (GR)—act as the first line of defense against free-radical-induced damage [54]. On the other hand, there is growing interest in natural anti-oxidants from plants, which are considered safer alternatives to synthetic compounds such as butylated hydroxyanisole (BHA) and butylated hydroxytoluene (BHT) [55]. In this context, the anti-oxidant potential of the four plant extracts was evaluated using multiple assays, as summarized in Table 4. Plant extracts have demonstrated significant anti-oxidant activity across various assays, including 2,2-diphenyl-1-picrylhydrazyl (DPPH), 2,2′-azinobis-(3-ethylbenzothiazoline-6-sulfonic acid) (ABTS), hydrogen peroxide (H_2_O_2_) scavenging, ferric reducing anti-oxidant power (FRAP), and cupric ion reducing anti-oxidant capacity (CUPRAC). A study on *Silybum marianum* reported that both aqueous and methanolic extracts obtained from different plant parts exhibited high anti-oxidant activity, particularly showing strong DPPH radical scavenging activity and pronounced anti-oxidant capacity in the ABTS assay [56]. Similarly, another study investigating the methanolic extract of *Picnomon acarna* demonstrated strong anti-oxidant activity across all tested assays, with IC_50_ values of 144.28 ± 0.16 μmol/g DW for DPPH, 81.57 ± 0.09 μmol/g DW for FRAP, and 85.57 ± 0.88 μmol/g DW for CUPRAC [57].

In addition, the same type of extract obtained from different parts of a plant may exhibit varying anti-oxidant activities and distinct IC_50_ values when evaluated using the same assay. For example, a study investigating the anti-oxidant activity of methanolic extracts of *Carduus pycnocephalus* derived from different plant parts (flowers, stems, roots, and leaves) demonstrated significant variation in activity using the DPPH assay. Among these, the methanolic extract of the flowers showed the strongest anti-oxidant potential, with the lowest IC_50_ value (32.78 mg/L) [45]. These differences are primarily attributed to variations in the total phenolic, flavonoid, and tannin contents among the different plant parts [45].

### 7.3. Anti-Cancer Activity

#### 7.3.1. Anti-Cancer Activity of *Silybum marianum*

*Silybum marianum* and its bioactive flavonolignans exhibit significant anti-cancer potential across various in vitro and in vivo models. The plant-derived compounds demonstrate strong anti-proliferative effects, induction of apoptosis, and modulation of key molecular signaling pathways involved in cancer progression [60]. These effects are largely mediated through the downregulation of major pathways such as PI3K/Akt, ERK, and Wnt/β-catenin, alongside inhibition of cell proliferation markers and activation of apoptotic mechanisms [61]. Overall, these findings highlight the therapeutic potential of *Silybum marianum* constituents as promising candidates for cancer treatment and drug development as shown in Table 5.

#### 7.3.2. Anti-Cancer Activity of *Carduus pycnocephalus*

*Carduus pycnocephalus* crude methanolic extract demonstrates notable cytotoxic activity against cancer cell lines, indicating its potential as a source of anti-cancer agents. The observed effect suggests a dose-dependent inhibition of tumor cell viability, reflecting the presence of bioactive compounds with anti-proliferative properties. Although the activity is considered moderate, it highlights the plant’s relevance in cancer research and the need for further phytochemical and mechanistic investigations. Overall, these findings support the potential application of *Carduus pycnocephalus* in developing novel therapeutic strategies against cancer, as shown in Table 6.

#### 7.3.3. Anti-Cancer Activity of *Glebionis segetum*

*Glebionis segetum* ethanolic extract exhibits low cytotoxic activity against the tested cancer cell lines, indicating limited anti-proliferative potential under the studied conditions. The extract showed weak or negligible effects on cell viability, suggesting that its bioactive constituents may not be sufficiently potent or present in effective concentrations [60]. These findings imply that the plant, in this form, may not be a strong candidate for anti-cancer applications compared to other medicinal species. Nevertheless, further studies focusing on different extraction methods or isolated compounds may reveal enhanced biological activity as shown in Table 7.

### 7.4. Anti-Diabetic, Anti-Enzymatic, and Anti-Obesity Activities

*Silybum marianum* exhibits a wide range of metabolic and enzyme-modulating activities, particularly in anti-diabetic, anti-obesity, and enzyme inhibition applications [67]. Various extracts and isolated compounds demonstrate strong inhibitory effects against key metabolic enzymes such as α-amylase, α-glucosidase, lipase, and PTP1B, indicating their potential in regulating glucose and lipid metabolism. In vivo studies further confirm significant hypoglycemic, anti-obesity, and metabolic regulatory effects, including reductions in body weight, serum glucose, and lipid-related parameters, alongside improvements in insulin levels and metabolic signaling pathways [68]. Additionally, its bioactive constituents show inhibitory activity against oxidative and inflammatory enzymes, supporting its therapeutic potential in managing metabolic disorders and related complications as shown in Table 8.

### 7.5. Anti-Inflammatory Activity

*Picnomon acarna* extracts demonstrate notable anti-inflammatory potential through the inhibition of tumor necrosis factor-alpha (TNF-α) in human monocytes. Both ethanolic and methanolic extracts exhibit significant inhibitory effects, indicating the presence of bioactive compounds capable of modulating inflammatory responses [61]. The ethanolic extract shows comparatively higher inhibitory activity, suggesting that solvent choice influences the extraction efficiency of active constituents. Overall, these findings support the potential of *Picnomon acarna* as a promising natural source of anti-inflammatory agents, as shown in Table 9.

*Silybum marianum* exhibits potent anti-inflammatory activity through multiple molecular and cellular mechanisms in both in vitro and in vivo models [79]. Its extracts and bioactive compounds effectively suppress key inflammatory mediators, including TNF-α, IL-6, nitric oxide, and reactive oxygen species, while also inhibiting pro-inflammatory enzymes such as COX, LOX, and sPLA2 [80]. Additionally, the plant demonstrates strong in vivo efficacy by reducing inflammation-related parameters in various disease models, including edema, nephrotoxicity, and ulcerativeolitis [81]. Overall, these findings highlight its significant therapeutic potential as a natural anti-inflammatory agent targeting multiple signaling pathways as shown in Table 10.

*Carduus pycnocephalus* L. demonstrates significant anti-inflammatory activity in formalin-induced rat paw edema models. Various extracts from the aerial parts show different levels of edema inhibition, with chloroform and ethyl acetate extracts producing the highest effect at over 75% inhibition [45]. Aqueous and butanol extracts also display substantial activity, while acetonitrile, ethanol, and petroleum ether extracts exhibit moderate to lower inhibition. These results indicate that the choice of solvent strongly influences the anti-inflammatory potency of *Carduus pycnocephalus* L. [25], highlighting its potential as a source of natural anti-inflammatory agents, as shown in Table 11.

## 8. Applications in Green Nanotechnology

**Figure 3:**
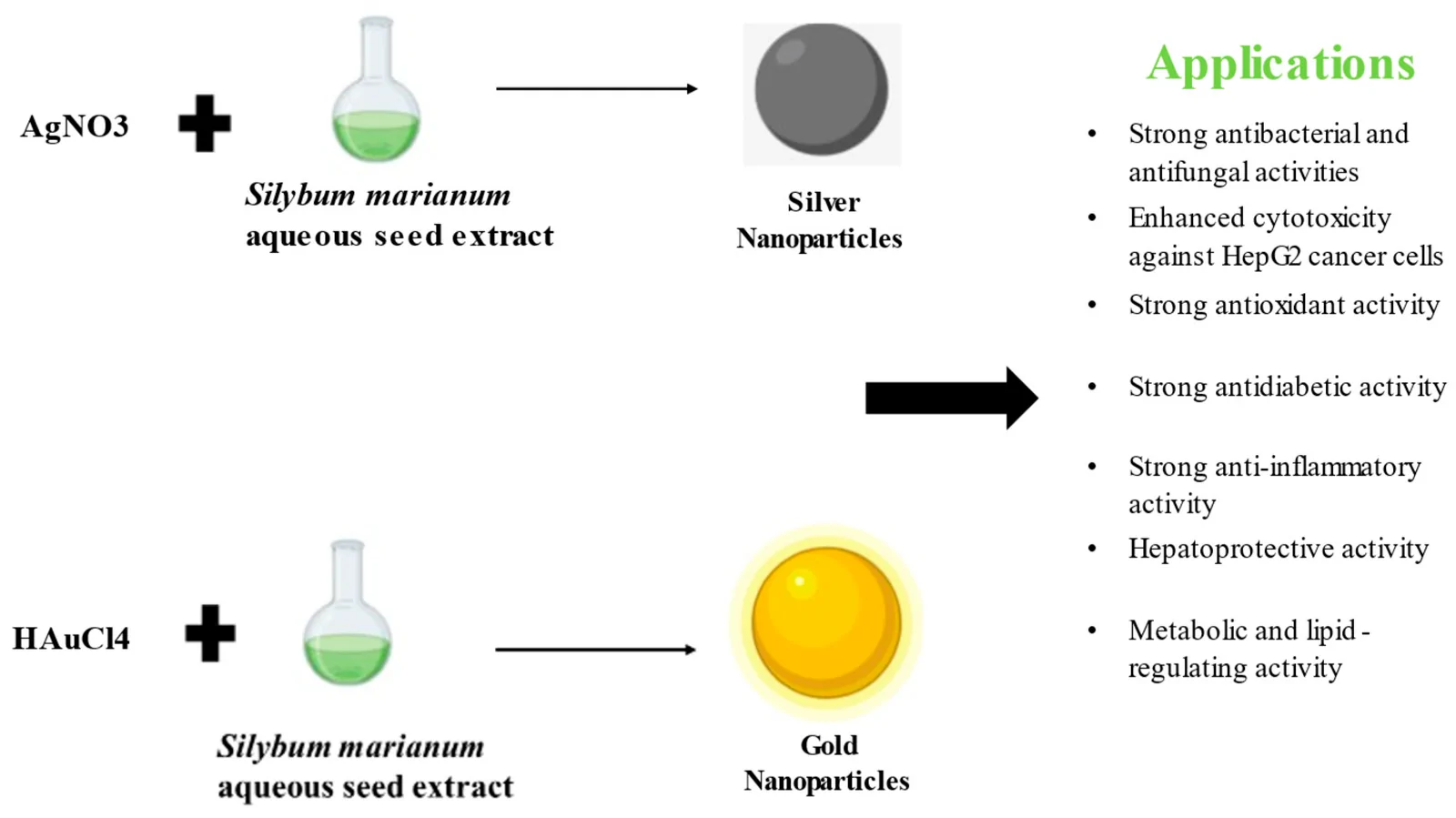
Reported therapeutic applications of silver and gold nanoparticles synthesized using *Silybum marianum* extract.

Nanotechnology is a rapidly advancing field with significant potential to impact multiple disciplines. It focuses on the manipulation of materials at the nanoscale (1–100 nm), where they display distinct physical, chemical, and biological characteristics compared to their bulk counterparts [90]. Nanoparticles can be produced through three primary approaches: physical, chemical, and biological methods [91]. Compared to conventional physical and chemical techniques, green synthesis offers several advantages in accordance with the principles of green chemistry [92]. This approach utilizes natural compounds obtained from plants or microorganisms—including fungi, bacteria, and algae—to reduce metal ions such as gold [93]. Plant extracts, which are rich in bioactive constituents such as flavonoids, terpenoids, amino acids, aldehydes, and alcohols, function as both reducing and stabilizing agents due to their strong redox potential [94]. Accordingly, extracts from *Silybum marianum*, a member of the Asteraceae family, have been successfully employed in the synthesis of gold and silver nanoparticles (Figure 3).

Silver nanoparticles are widely applied as anti-microbial agents in the medical field because of their potent inhibitory activity against a broad range of microorganisms, including bacteria, fungi, and viruses, while exhibiting relatively low toxicity toward human cells [95]. A study reported the synthesis of silver nanoparticles using aqueous seed extract of *Silybum marianum* as both reducing and capping agents [96]. The synthesized silver nanoparticles demonstrated strong anti-oxidant activity [97]. Moreover, enhanced anti-bacterial effects were observed against *Klebsiella pneumonia*, *Staphylococcus aureus*, *Escherichia coli*, and *Enterobacter intermedius*, along with anti-fungal activity against *Candida albicans* [98].

On the other hand, gold nanoparticles have been synthesized from *Silybum marianum*. A study conducted by Clichici et al. showed that the synthesized gold nanoparticles reduced oxidative stress, as evidenced by decreased MDA levels, and exhibited anti-fibrotic and anti-inflammatory effects, reflected by lowered α-SMA expression and TGF-β1 levels, along with reduced pNF-κB levels, indicating significant anti-inflammatory activity [99]. Table 12 presents a summary of nanoparticles synthesized using *Silybum marianum*, including their physicochemical properties and biological activities.

## 9. Other Applications

The four Asteraceae species reviewed herein exhibit considerable potential as sources of functional foods, nutraceuticals, and medicinal products [102]. Nevertheless, the current body of evidence reveals marked disparities in both their utilization and scientific characterization [103]. While *Picnomon acarna*, *Carduus pycnocephalus*, and *Glebionis segetum* have primarily been recognized as traditional edible plants in Mediterranean cultures, *Silybum marianum* has progressed substantially beyond ethnobotanical use, supported by extensive investigations into its nutritional properties, food applications, and toxicological safety [38,104]. This contrast illustrates a common trend in medicinal plant research, where longstanding traditional use often precedes comprehensive scientific validation. Therefore, integrating food application studies with toxicological evidence provides a more balanced perspective on the translational potential of these species for functional food and nutraceutical development [105]. *Picnomon acarna* has a long history of consumption as a traditional wild edible vegetable throughout the Mediterranean basin [106]. Ethnobotanical surveys from Lebanon indicate that both its raw leaves and roots are commonly consumed in salads prepared with olives, onions, and bread, reflecting its cultural importance in rural communities [107]. Similar reports across the Mediterranean identify *P. acarna* as one of several wild Asteraceae species traditionally harvested as seasonal vegetables, contributing to dietary diversity and local food security [108]. In Balkan countries, its roots are cooked as vegetables, while floristic references classify the species as a traditional European potherb and recognize it as an edible wild plant even beyond its native distribution [109]. Despite this extensive traditional use, scientific studies addressing its nutritional composition, bioavailability of phytochemicals following culinary preparation, and long-term safety remain scarce [110]. Moreover, no experimental investigations have systematically evaluated its acute or chronic toxicity, organ-specific effects, genotoxicity, reproductive toxicity, or allergenic potential. Consequently, although centuries of traditional consumption suggest an acceptable safety profile, the absence of rigorous toxicological evidence represents a major limitation that currently restricts its development as a scientifically validated functional food or nutraceutical ingredient [111]. Future investigations should therefore integrate nutritional characterization with standardized toxicological assessment to facilitate its safe commercialization. Among the four species, *Silybum marianum* represents the most advanced example of successful translation from traditional medicine to modern functional food applications [71]. Recent studies have demonstrated that milk thistle seed flour can be incorporated into bakery products, significantly improving dietary fiber content, anti-oxidant capacity, and overall nutritional quality without compromising sensory acceptability [112]. Similar benefits have been observed in gluten-free formulations, where partial substitution of rice flour with milk thistle flour enhanced nutritional value while maintaining desirable technological properties. In addition to flour applications, milk thistle seeds constitute a valuable source of edible vegetable oil characterized by favorable fatty acid composition, high oxidative stability, and abundant bioactive constituents, including phenolic compounds and silymarin derivatives [113]. Comparative analyses of different genotypes further demonstrate considerable variability in oil yield, essential fatty acid composition, and phytochemical content, highlighting opportunities for cultivar selection aimed at maximizing nutritional quality and commercial value. Importantly, the development of *S. marianum* as a functional food ingredient has been supported by extensive toxicological investigations demonstrating an excellent safety profile [74]. Clinical and experimental studies consistently report high tolerability at therapeutic doses, with only mild and transient gastrointestinal adverse effects documented in most cases. Although high-dose silibinin formulations have occasionally produced reversible elevations in hepatic enzymes, the overall evidence indicates a wide margin of safety, with oral LD_50_ values exceeding 2000 mg/kg in experimental animals and no evidence of significant cytotoxicity or chromosomal damage in vivo [114]. Nevertheless, reports of reproductive toxicity at very high doses in animal models suggest that cautious use during pregnancy remains advisable until more comprehensive human data become available. Collectively, *S. marianum* represents the most scientifically validated species among those reviewed, providing a successful model for the translation of traditional medicinal plants into evidence-based nutraceutical and functional food products [19].

*Carduus pycnocephalus*, likewise, occupies an important place in Mediterranean food traditions, where its young leaves are consumed raw or cooked, and its tender stems are eaten fresh as seasonal foraged vegetables [115]. Scientific investigations have confirmed that this species contains appreciable concentrations of phenolic compounds and flavonoids that contribute to its anti-oxidant capacity and nutritional value. These phytochemical characteristics support its potential as a functional food resource; however, research remains largely descriptive, with limited information regarding nutrient bioavailability, processing stability, consumer acceptance, or industrial food applications [116]. Toxicological investigations are similarly limited. However, preclinical safety assessments in female mice concluded that the hydroalcoholic extract of *Carduus pycnocephalus* is relatively safe, exhibiting an LD50 greater than 2000 mg/kg in acute oral studies and showing no significant biochemical or histopathological toxicity in repeated-dose trials at levels below 200 mg/kg [36]. Similarly, *Glebionis segetum* has traditionally been consumed throughout Mediterranean countries as a cooked leafy vegetable and has recently attracted growing attention because of its edible flowers, which combine attractive sensory characteristics with considerable anti-oxidant and polyphenolic content [107]. Studies indicate that the flowers maintain satisfactory quality during refrigerated storage, supporting their potential use in salads, garnishes, and other culinary applications. Furthermore, broader evaluations of edible flowers identify *G. segetum* as an emerging species of gastronomic interest whose nutritional and sensory quality is influenced by genotype, environmental conditions, and cultivation practices [117]. Despite these promising characteristics, food-related investigations remain preliminary and have yet to evaluate processing technologies, shelf-life optimization, consumer acceptance, or commercial feasibility [118]. Toxicological evidence is even more limited, with available studies focusing primarily on its insecticidal activity rather than mammalian safety. Chronic exposure produces growth inhibition, prolonged larval development, and increased mortality in *Spodoptera littoralis*, indicating the presence of biologically active metabolites capable of disrupting physiological processes [119]. While these findings support the plant’s potential as a natural bioinsecticide, their implications for human safety remain uncertain and warrant dedicated toxicological evaluation [120]. Overall, comparison of the four species reveals a clear disparity between traditional utilization and scientific validation. Whereas *S. marianum* has progressed toward commercial functional food and nutraceutical applications through extensive investigations of both efficacy and safety, *P. acarna*, *C. pycnocephalus*, and *G. segetum* remain largely supported by ethnobotanical knowledge and preliminary phytochemical studies [121,122]. Their long histories of traditional consumption undoubtedly support their potential as valuable dietary resources; however, traditional use alone cannot substitute for comprehensive nutritional, pharmacological, and toxicological evaluation. Future research should therefore adopt multidisciplinary approaches integrating food science, phytochemistry, toxicology, and clinical nutrition to establish standardized quality parameters, determine the bioavailability of bioactive compounds following food processing, evaluate long-term safety, and define evidence-based recommendations for their incorporation into modern functional foods and nutraceutical formulations.

**Figure 4:**
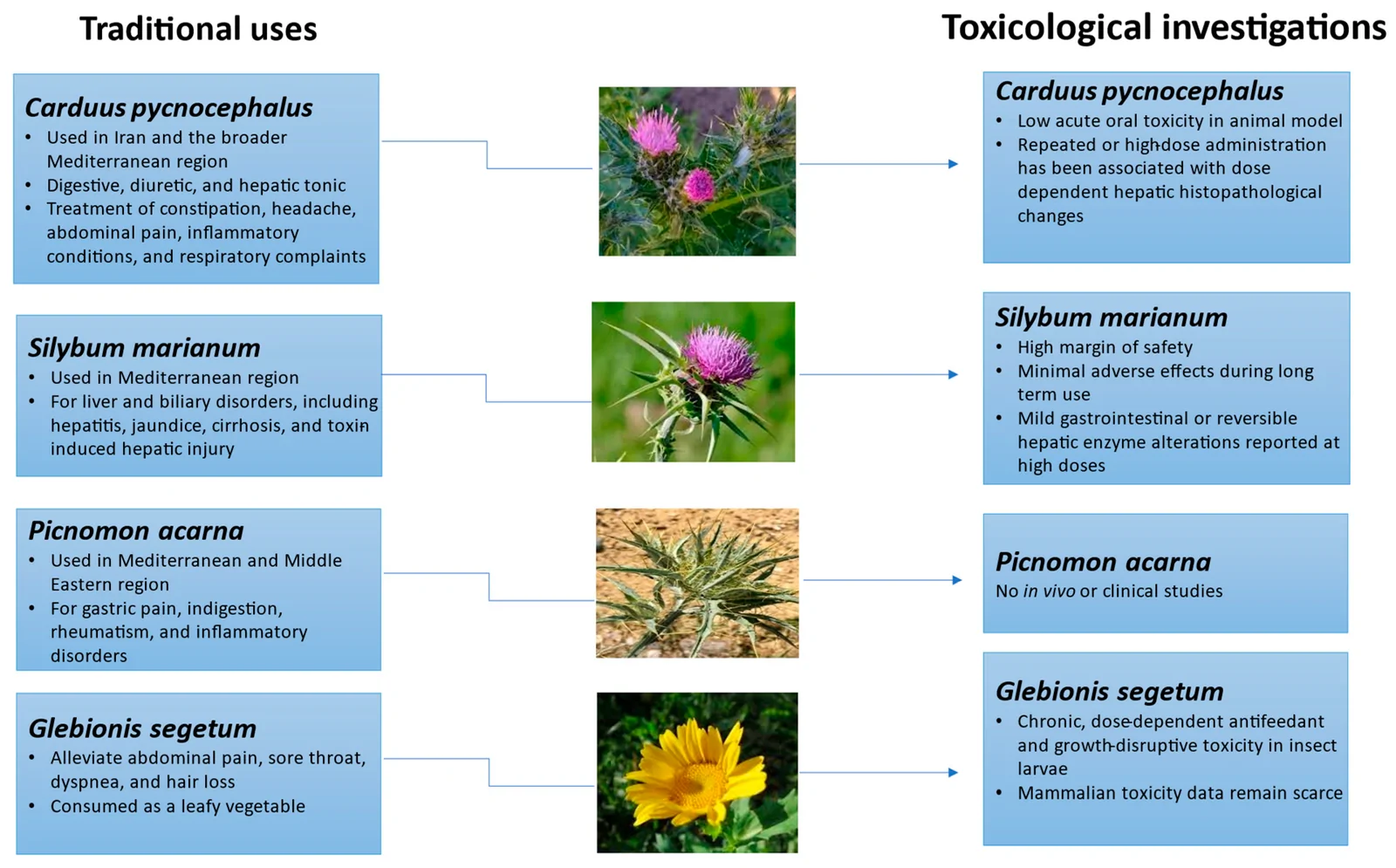
The traditional uses and the toxicological investigation of the studied species.

The traditional uses in addition to the toxicological properties of the four species studied are illustrated in Figure 4.

## 10. Conclusions

*Picnomon acarna*, *Silybum marianum*, *Carduus pycnocephalus*, and *Glebionis segetum* are invasive yet highly adaptive species of the Asteraceae family, widely distributed across Mediterranean regions. These plants have long been utilized in traditional medicine; for example, *P. acarna* is used for digestive disorders, *S. marianum* for liver diseases, *C. pycnocephalus* for inflammatory conditions, and *G. segetum* for sore throat. Their medicinal value is largely attributed to their richness in bioactive phytochemicals such as flavonoids, tannins, and phenolic compounds.

Beyond these traditional uses, extracts from these species have demonstrated a broad spectrum of biological activities, including anti-bacterial, anti-fungal, anti-oxidant, anti-cancer, anti-diabetic, anti-enzymatic, anti-obesity, and anti-inflammatory effects, highlighting their significant therapeutic potential. In addition, these plant extracts have been successfully employed as reducing and capping agents in the green synthesis of gold and silver nanoparticles, which exhibit diverse biomedical applications. Furthermore, some of these species are increasingly incorporated into modern food systems as sources of nutrient-rich flour and edible oils, contributing to both nutritional diversity and sustainable food practices. Overall, their multifunctional biological properties continue to attract considerable research interest, highlighting their potential as promising candidates for the development of future therapeutic applications. However, further investigations, including pharmacological studies and clinical trials, are required to validate their efficacy and therapeutic potential.

Despite these promising findings, most studies remain limited to crude extracts and in vitro investigations, highlighting the need for bioassay-guided isolation and characterization of the active compounds, followed by mechanistic, in vivo, and clinical studies to validate their therapeutic potential. In particular, the substantial hepatoprotective evidence for *S. marianum* provides a strong basis for progression to well-designed controlled clinical trials. Comprehensive toxicological and pharmacokinetic evaluations are also required, especially for *P. acarna*, *C. pycnocephalus*, and *G. segetum*, to establish their safety profiles and therapeutic dosage ranges. Future research should further optimize the green synthesis of nanoparticles for applications in drug delivery, biosensing, and other biomedical technologies, while also assessing the long-term safety and efficacy of these species as functional food ingredients and nutraceuticals. Ultimately, sustainable valorization strategies that transform these invasive plants into valuable pharmaceutical, nutraceutical, and biotechnological resources, supported by interdisciplinary collaboration among phytochemists, pharmacologists, toxicologists, and biotechnologists, will be essential for translating their traditional uses and biological potential into safe, evidence-based applications.

## Figures and Tables

**Table 1:** Summary of the phytochemical constituents identified in extracts from different parts of *Picnomon acarna*, *Silybum marianum*, *Carduus pycnocephalus*, and *Glebionis segetum* using various solvents.

Plant Name	Plant Part Extract	Extract Type	Technique Used for Identification	Phytochemical Names	References
*Picnomon acarna*	Leaves and flowers	Fractionation: Ethyl acetate fraction	TLC + PC	• Gossypetin-8,3′,4′-trimethyl ether • luteolin-7,3′-dimethyl ether • luteolin-3′-methyl ether	[41]
*Picnomon acarna*	Areial parts of the plant	Fractionation of Methanolic extract: Diethyl ether Ethyl acetate n-Butanol	^1^H-NMR EIMS UV NOESY	• Coumarins: 6,7,8-Trimethoxycoumarin • Flavonoid aglycones Hispidulin Luteolin Quercetin 5,4′-Dihydroxy-6-methoxyflavone • Flavonoid glycosides Pectolinarin Linarin Homoplantagin Hyperin 5,4′-Dihydroxy-6-methoxyflavone-7-O α-L-rhamnopyranoside • Phenolic acids/Phenolic glycosides Gentisic acid 5-O-glucoside	[42]
*Silybum marianum*	Whole plant	Ethanol/water extract	LC-MS/MS	33 types including: Flavonolignans (silybin, isosilybin, silychristin, etc.), phenolics, flavonoids, tannins	[43]
*Silybum marianum*	Flowers	Methanol/water extract	• Chromatographic techniques: PC + TLC • Spectroscopic techniques: UV–Vis ^1^H + ^13^C NMR	• apigenin 7-O-β-(2″-O-α-rhamnosyl) galacturonide • kaempferol 3-O-α-rhamnoside-7-O-β-galacturonide • apigenin 7-O-β-glucuronide 6″-ethyl ester • apigenin 7-O-β-glucoside • apigenin 7-O-β-galactoside • kaempferol-3-O-α-rhamnoside • kaempferol	[44]
*Silybum marianum*	Seeds oil	Soxhlet petroleum-ether extraction	HPLC-UV	• Unsaturated fatty acids: Linoleic acid, Oleic acid • Phytosterols: Campesterol, β-Sitosterol, Stigmasterol, β-Amyrin • Tocopherols: α-Tocopherol • Phenolic compounds	[42]
*Carduus pycnocephalus*	Whole plant	Methanolic extract	GC–MS	• 2-(Hept-6-yn-1-yl) malonic acid • (E)-4-(((2-Methoxyoctadec-4-en-1-yl)oxy)methyl) -2,2-dimethyl-1,3-dioxolane • 3,5-Dihydroxy-6-methyl-2,3-dihydro-4H-pyran-4-one • Methyl 11-((2R,3S)-3-pentyloxiran-2-yl) undecanoate • Methyl (E)-octadec-11-enoate • Ethyl iso-allocholate	[45]
*Carduus pycnocephalus*	Aerial parts	• Essential oil • Petroleum ether extract → unsaponifiable fraction + Fatty acid methyl esters (FAMEs)	GC/MS	• Essential oil: 19 compounds, major ones: Hexadecanoic acid + 9,12-Linoleic acid • Unsaponifiable matter: 16 compounds, major ones: Olean-12-en-3-ol + Ursa-9(11),12-dien-3-ol • FAMEs: 20 fatty acids, major ones: 1,2-Benzenedicarboxylic acid, dimethyl ester + Palmitic acid	[45]
*Carduus pycnocephalus*	Aerial parts	Ethanolic extract + fractionation: petroleum ether → chloroform → ethyl acetate → butanol → water	• Chromatographic techniques: silica gel column chromatography + TLC • Spectroscopic techniques: UV Spectroscopy + IR + NMR + MS	Flavonoids + sterols: • apigenin • kaempferol-3-O-β-d-glucoside • kaempferol-3-O-α-l-rhamnoside • kaempferol-7-methoxy-3-O-α-l-rhamnoside • β-sitosterol • β-sitosterol-3-O-β-d-glucoside • Lupeol • Diosmetin-7-O-β-D-xylosyl-(1 → 6)-β-D-glucopyranoside • Diosmetin-7-O-α-L-arabinosyl-(1 → 6)-β-D-glucopyranoside • Kaempferol-3-O-α-L-rhamnosyl-(1 → 2)-α-L-rhamnoside	[25]
*Carduus pycnocephalus*	Aerial parts	Ethanolic extract + fractionation: Petroleum ether → Chloroform → Ethyl acetate → butanol → Aqueous	• Chromatographic techniques: TLC + HPLC • Spectroscopic techniques: UV + MS + NMR	• 3-O-acetyl-ursolic acid-28-ethyl ester • Bis (2-ethylhexyl) benzene-1,2-dicarboxylate • 3α, 24-dihydroxyolean-12-en-28, 30-dioic acid dimethyl ester • Kaempferol • Diosmetin-7-O-α-L-arabinopyransyl (1‴ → 4″)-β-D-glucopyranoside	[25]
*Glebionis segetum*	Aerial parts	Essential oil	GC–MS	28 compounds, major ones: • Capillene • caryophyllene oxide • 1-phenyl-penta-2,4-diyne • capillin	[26]
*Glebionis coronaria*	Aerial parts	Essential oil	GC–MS	9 compounds, major ones: • Capillin • Capillene • caryophyllene oxide	[26]

**Table 2:** Anti-microbial activity of *Silybum marianum*.

Plant Name	Solvent used for Extraction	Plant Part	Dose Used	Experimental Model	Results	References
*Silybum marianum*	Ethanol (95%)	Seeds	600 mg/mL	Agar-well diffusion against -*Staphylococcus aureus* (MRSA) -*Stenotrophomonas maltophilia* -*Klebsiella pneumonia* -*Escherichia coli*	Inhibition zones (mm): -MRSA: 33.67 -*S. maltophilia*: 34.33 -*K. pneumoniae*: 25.00 -*E. coli*: 21.00	[50]
*Silybum marianum*	Acetone	Seeds	600 mg/mL	Agar-well diffusion against -*Staphylococcus aureus* (MRSA) -Gram-negative bacteria	Inhibition zone (mm): -MRSA: 25.33 -No activity against Gram-negative bacteria.	[50]
*Silybum marianum*	Cold Aqueous (Water)	Seeds	600 mg/mL	Agar-well diffusion against -Gram-positive and Gram-negative bacteria	Inhibition zones (mm): -*S. maltophilia*: 20.00 -*E. coli*: 14.33 -*K. pneumoniae*: 11.67 -No activity against MRSA.	[50]
*Silybum marianum*	Hot Aqueous (Water)	Seeds	N/A	Agar-well diffusion against MDR bacteria.	No anti-microbial activity was observed against any of the tested MDR strains.	[50]
*Silybum marianum*	Chloroform	Seeds (from flowers)	Various	Disk diffusion and Broth macro-dilution (MIC/CMB): -*Staphylococcus aureus* -*Staphylococcus albus* -*Candida albicans* -*Saccharomyces cerevisiae*	Action was determined to be bacteriostatic. Disk diffusion: Zones of 17–18 mm for *S. aureus* and *S. albus*; 9–16 mm for fungi. MIC: 10.25–20.5 mg/mL CMB: 41 mg/mL	[51]
*Silybum marianum*	n-Butanol	Seeds (from flowers)	Various	Disk diffusion and Broth macro-dilution (MIC/CMB): -*Staphylococcus aureus* -*Staphylococcus albus* -*Candida albicans* -*Saccharomyces cerevisiae*	Action was determined to be bacteriostatic. Disk diffusion: Zones of 17–18 mm for *S. aureus* and *S. albus*; 9–16 mm for fungi. MIC: 7–14 mg/mL CMB: 28 mg/mL	[51]
*Silybum marianum*	Hydroalcoholic (Ethanol) (Ultrasound-assisted extraction)	Crude extract of a whole plant	12.5, 25, and 50 mg/mL	Agar disk diffusion: -*Staphylococcus aureus* -*Escherichia coli* -*Aspergillus oryzae* -*Candida albicans*	Inhibition zones (mm) at 50 mg/mL: -*S. aureus*: 12.30 -*E. coli*: 11.38 -*C. albicans*: 7.68 -*A. oryzae*: 7.44	[49]
*Silybum marianum*	Methanol	Flowers	100 µL	Agar-well diffusion: -*B. subtilis*, *S. aureus*, *E. coli*, *P. aeruginosa*, *S. typhimurium*, *C. albicans*	Inhibition zones (mm): 21.33, 26.33, 13.1, 24.96, 10, 17.13	[50]
*Silybum marianum*	Methanol	Leaves	100 µL	Agar-well diffusion: -*B. subtilis*, *S. aureus*, *E. coli*, *P. aeruginosa*, *S. typhimurium*, *C. albicans*	Inhibition zones (mm): 16.4, 17.4, 13.5, 24.6, 13.3, 13.8	[50]
*Silybum marianum*	Methanol	Stems	100 µL	Agar-well diffusion: -*B. subtilis*, *S. aureus*, *E. coli*, *P. aeruginosa*, *S. typhimurium*, *C. albicans*	Inhibition zones (mm): 21.0, 17.83, 22.1, 32.16, 18.16, 14.16	[50]
*Silybum marianum*	Acetone	Flowers	100 µL	Agar-well diffusion: -*B. subtilis*, *S. aureus*, *E. coli*, *P. aeruginosa*, *S. typhimurium*, *C. albicans*	Inhibition zones (mm): 23.3, 27.3, 12.2, 24.7, 14, 18	[50]
*Silybum marianum*	Acetone	Leaves	100 µL	Agar-well diffusion: -*B. subtilis*, *S. aureus*, *E. coli*, *P. aeruginosa*, *S. typhimurium*, *C. albicans*	Inhibition zones (mm): 17.4, 18.4, 15.2, 24, 23, 12.8	[50]
*Silybum marianum*	Acetone	Stems	100 µL	Agar-well diffusion: -*B. subtilis*, *S. aureus*, *E. coli*, *P. aeruginosa*, *S. typhimurium*, *C. albicans*	Inhibition zones (mm): 15.7, 19.5, 23.3, 29.5, 12.3, 13.2	[50]
*Silybum marianum*	Ethyl Acetate	Flowers	100 µL	Agar-well diffusion: -*B. subtilis*, *S. aureus*, *E. coli*, *P. aeruginosa*, *S. typhimurium*, *C. albicans*	Inhibition zones (mm): 22.3, 13.1, 23.4, 23.7, 12.8, 12.1	[50]
*Silybum marianum*	Ethyl Acetate	Leaves	100 µL	Agar-well diffusion: -*B. subtilis*, *S. aureus*, *E. coli*, *P. aeruginosa*, *S. typhimurium*, *C. albicans*	Inhibition zones (mm): 18.6, 13.4, 14.1, 23.4, 22, 10	[50]
*Silybum marianum*	Ethyl Acetate	Stems	100 µL	Agar-well diffusion: -*B. subtilis*, *S. aureus*, *E. coli*, *P. aeruginosa*, *S. typhimurium*, *C. albicans*	Inhibition zones (mm): 13, 17.07, 23.9, 28.4, 13.1, 14.17	[50]
*Silybum marianum*	Petroleum Ether	Flowers	100 µL	Agar-well diffusion: -*B. subtilis*, *S. aureus*, *E. coli*, *P. aeruginosa*, *S. typhimurium*, *C. albicans*	Inhibition zones (mm): 22, 23.53, 18.43, 21.33, 10, 10	[50]

**Table 3:** Anti-microbial activities of *Carduus pycnocephalus*.

Plant Name	Solvent Used for Extraction	Plant Part	Dose Used	Experimental Model	Results	References
*Carduus pycnocephalus* L.	Methanol	Root	10 mg/L	Disk diffusion -Gram-negative: *E. coli*, *P. aeruginosa*, *S. typhimurium*, *S. epidermidis* -Gram-positive: *B. cereus*, *S. aureus*, *S. haemolyticus*, *S. xylosus*, *K. pneumonius*	Inhibition zones (mm): -*P. aeruginosa*: 22 -*S. typhimurium*: 14 -*E. coli*: 13 -*S. aureus*: 12 -*B. cereus*: 11 -*S. epidermidis* and *K. pneumonius*: 10 -No activity against *S. haemolyticus* or *S. xylosus*	[45]
*Carduus pycnocephalus* L.	Methanol	Stem	10 mg/L	Disk diffusion -Gram-negative: *E. coli*, *P. aeruginosa*, *S. typhimurium*, *S. epidermidis* -Gram-positive: *B. cereus*, *S. aureus*, *S. haemolyticus*, *S. xylosus*, *K. pneumonius*	Inhibition zones (mm): -*P. aeruginosa*: 20 -*S. typhimurium* and *B. cereus*: 13 -*E. coli*, *S. aureus*, *K. pneumonius*: 10 -*S. epidermidis*: 5 -No activity against *S. haemolyticus* or *S. xylosus*	[45]
*Carduus pycnocephalus* L.	Methanol	Leaf	10 mg/L	Disk diffusion -Gram-negative: *E. coli*, *P. aeruginosa*, *S. typhimurium*, *S. epidermidis* -Gram-positive: *B. cereus*, *S. aureus*, *S. haemolyticus*, *S. xylosus*, *K. pneumonius*	Inhibition zones (mm): -*S. typhimurium*: 26 -*B. cereus*: 23 -*E. coli*: 20 -*K. pneumonius*: 13 -*P. aeruginosa* and *S. aureus*: 10 -*S. epidermidis* and *S. haemolyticus*: 5 -No activity against *S. xylosus*	[45]
*Carduus pycnocephalus* L.	Methanol	Flower	10 mg/L	Disk diffusion -Gram-negative: *E. coli*, *P. aeruginosa*, *S. typhimurium*, *S. epidermidis* -Gram-positive: *B. cereus*, *S. aureus*, *S. haemolyticus*, *S. xylosus*, *K. pneumonius*	Inhibition zones (mm): -*S. typhimurium* and *B. cereus*: 25 -*E. coli*: 20 -*S. epidermidis*: 15 -*S. aureus*: 14 -*K. pneumonius*: 13 -*P. aeruginosa*: 10 -*S. haemolyticus* and *S. xylosus*: 5	[45]
*Carduus pycnocephalus* L.	Ethanol (95%)	Aerial parts	50 µg/mL	Agar diffusion -Fungi: *Aspergillus fumigatus*, *Syncephalastrum racemosum*, *Geotricum candidum*, *Candida albicans* -Gram-positive: *Streptococcus pneumoniae*, *Bacillus subtilis* -Gram-negative: *Pseudomonas aeruginosa*, *Escherichia coli*	Inhibition zones (mm): -*G. candidum*: 18.6 -*B. subtilis*: 18.6 -*S. pneumonia*: 17.3 -*E. coli*: 17.2 -*A. fumigatus*: 16.8 -*P. aeruginosa*: 15.3 -*S. racemosum*: 15.2 -No activity against *C. albicans*	[45]
*Carduus pycnocephalus* L.	Essential oil	Aerial parts	5 mg/disk	Anti-microbial test resulting in minimum inhibitory concentration (MIC) -*Bacillus subtillus*, *Staphylococcus aereus*, *Mucobacterium smegmatus* -Also tested against: *Escherichia coli*, *Pseudomonas aeruginosa*, *Candida albicans*	MIC (mg/mL): -*Bacillus subtillus*: 1 -*Staphylococcus aereus*: 5 -*Mucobacterium smegmatus*: 5 -No activity against *E. coli*, *P. aeruginosa*, or *C. albicans*	[45]

**Table 4:** The anti-oxidant activities of *Picnomon acarna*, *Silybum marianum*, *Carduus pycnocephalus*, and *Glebionis segetum* extracts.

Plant Name	Solvent Used for Extraction	Plant Part	Dose Used	Methods Used	Results	References
*Picnomon acarna*	n-hexane extract+ chloroform/methanol extract	Seeds	Not reported	DPPH, FRAP, CUPRAC	• DPPH: 144.28 ± 0.16 μmol/g DW • FRAP: 81.57 ± 0.09 μmol/g DW • CUPRAC: 85.57 ± 0.88 μmol/g DW	[57]
*Silybum marianum*	Ethanol–water (microwave-enhanced extraction)	Whole plant	Not reported	• DPPH • ABTS • FRAP • CUPRAC • Phenanthroline	• DPPH IC_50_: 19.2 ± 2.3 µg/mL • ABTS IC_50_: 7.2 ± 1.7 µg/mL • FRAP IC_50_: 24.1 ± 1.2 µg/mL • CUPRAC IC_50_: 22.2 ± 1.2 µg/mL • Phenanthroline IC_50_: 35.2 ± 1.8 µg/mL	[58]
*Silybum marianum*	Water and ethanol	Leaves, roots, seeds	Concentration range (μg/mL): 5–100	• DPPH • ABTS	• DPPH IC_50_: Ethanolic extract: 8.53 ± 0.95–248.38 ± 7.10 μg/mL Aqueous extract: 689.14 ± 9.59–5578.62 ± 1378.93 μg/mL • ABTS IC_50_: Ethanolic extract: 64.56 ± 1.77–93.48 ± 4.74 μg/mL Aqueous extract: 78.09 ± 1.05–191.75 ± 9.12 μg/mL	[59]
*Silybum marianum*	Aqueous extract	Seeds + fruits	Concentration range (mg/mL): 10–30	• Ferric thiocyanate method • DPPH • ABTS • Ferrous ion (Fe^2+^) • H_2_O_2_ • Superoxide anion • Cupric ions (Cu^2+^)	• Ferric thiocyanate method: 82.7% inhibition of lipid peroxidation • DPPH: IC_50_ = 20.8 mg/mL • ABTS: EC_50_ = 8.62 mg/mL • Ferrous ion (Fe^2+^): 85.5% chelation • H_2_O_2_: 78.5% scavenging • Superoxide anion: 40.2% scavenging • Cupric ions (Cu^2+^): Concentration-dependent Cu^2+^ reducing capability	[59]
*Carduus pycnocephalus*	Methanol	Flowers	Concentration range (mg/L): 5–50 (serial dilutions: 5, 10, 20, 30, 40, 50 mg/L)	DPPH assay	IC_50_ (mg/L) 30.69	[25]
*Carduus pycnocephalus*	Methanol	Leaves	Concentration range (mg/L): 5–50 (serial dilutions: 5, 10, 20, 30, 40, 50 mg/L)	DPPH assay	IC_50_ (mg/L) 32.78	[25]
*Carduus pycnocephalus*	Methanol	Stems	Concentration range (mg/L): 5–50 (serial dilutions: 5, 10, 20, 30, 40, 50 mg/L)	DPPH assay	IC_50_ (mg/L) 41.31	[25]
*Carduus pycnocephalus*	Methanol	Roots	Concentration range (mg/L): 5–50 (serial dilutions: 5, 10, 20, 30, 40, 50 mg/L)	DPPH assay	IC_50_ (mg/L) 46.84	[25]
*Glebionis segetum*	Water (hydrodistillation)	Aerial parts	Concentration range (µg/mL): 0.49–250	DPPH assay	IC_50_: 2.525 ± 0.017 mg/mL	[26]

**Table 5:** Anti-cancer activity of *Silybum marianum*.

Plant Name	Solvent Used for Extraction	Plant Part	Dose Used	Experimental Model	Results	References
*Silybum marianum*	N/A	Commercial *Silibinin* flavonoid	In vitro: 50 µM. In vivo: 50 and 100 mg/kg	In vitro: Cell lines: Human ovarian cancer cells (A2780, SKOV3). Assays used: MTT assay, Annexin V staining, ROS measurement, Western blot (ERK, Akt). In vivo: Animal model: Balb/c nude mice with subcutaneous A2780 xenograft. Tests used: Tumor volume measurement, IHC (Ki-67, caspase-3, p-ERK, p-Akt), TUNEL assay.	In vitro: Decreased cell viability (58–65% cell viability). Induced apoptosis (42.09% in A2780; 26.19% in SKOV3). Caused downregulation of ERK and Akt. In vivo: Reduced tumor volume. Decreased cell proliferation (Ki-67-positive cells) and induced apoptosis (TUNEL-positive cells, caspase-3 activation). Inhibited p-ERK and p-Akt.	[62,63]
*Silybum marianum*	75% methanol-water (*v*:*v*) extract	Dried fruit (*Silydianin component*)	5–100 µM/L	In vitro: Screening tool: SNAP-tag-EGFR/CMC-HPLC-MS two-dimensional system. Cell lines: SNAP-tag-EGFR HEK293 (overexpressing EGFR) and NC-HEK293 (control). Verification tests: CCK-8 assay and nonlinear chromatography (NLC)	Inhibited proliferation of SNAP-tag-EGFR HEK293 cells in a dose-dependent manner. Affinity equilibrium constant KA = 347.78 ± 59.04 L/mol	[64]
*Silybum marianum*	75% methanol-water (*v*:*v*) extract	Dried fruit (*Silychristin)*	5–100 µM/L	In vitro: Screening tool: SNAP-tag-EGFR/CMC-HPLC-MS two-dimensional system. Cell lines: SNAP-tag-EGFR HEK293 (overexpressing EGFR) and NC-HEK293 (control). Verification tests: CCK-8 assay and NLC	Inhibited proliferation of SNAP-tag-EGFR HEK293 cells in a dose-dependent manner. Affinity equilibrium constant KA = 685.76 ± 169.15 L/mol.	[64]
*Silybum marianum*	75% methanol-water (*v*:*v*) extract	Dried fruit (*Silybin*)	5–100 µM/L	In vitro: Screening tool: SNAP-tag-EGFR/CMC-HPLC-MS two-dimensional system. Cell lines: SNAP-tag-EGFR HEK293 (overexpressing EGFR) and NC-HEK293 (control). Verification tests: CCK-8 assay and NLC.	Inhibited proliferation of SNAP-tag-EGFR HEK293 cells in a dose-dependent manner. Affinity equilibrium constant KA = 2025.62 ± 405.94 L/mol.	[64]
*Silybum marianum*	75% methanol-water (*v*:*v*) extract (screened/isolated compound)	Dried fruit (*Isosilybin*)	5–100 µM/L	In vitro: Screening tool: SNAP-tag-EGFR/CMC-HPLC-MS two-dimensional system. Cell lines: SNAP-tag-EGFR HEK293 (overexpressing EGFR) and NC-HEK293 (control). Verification tests: CCK-8 assay and NLC	Inhibited proliferation of SNAP-tag-EGFR HEK293 cells in a dose-dependent manner. Most potent inhibitory effect among the four compounds tested. Affinity equilibrium constant KA = 2227.62 ± 152.09 L/mol.	[64]
*Silybum marianum*	Water extract	Seed	In vitro: 5 mg/mL, 10 mg/mL. In vivo: 0.5%, 1%, 1.5% (oral feeding).	In vitro: Cell lines: Human breast cancer cells (T-47D, ZR-751, MDA-MB-231). Assays Used: MTT assay. Western blot (PLK1, EXTAH complex proteins). In vivo: Animal model: Balb/c mice orthotopically injected with 4T1 cells. Assay used: IHC (PCNA, PLK1).	In vitro: Decreased cell viability dose- and time-dependently. Increased mitosis disruption through downregulation of PLK1 and associated spindle assembly proteins (TPX2, Eg5, HURP). In vivo: Significantly decreased tumor growth. Reduced PCNA and PLK1 expression in tumor specimens.	[65]
*Silybum marianum* Total Extract (STE)	Methanol and Petroleum ether extraction	Fruits	In vitro: 0–250 µg/mL. In vivo: 200 mg/kg bw (oral, every other day).	In vitro: Cell lines: Human hepatocellular carcinoma cell lines (HepG2, Huh7). Assay Used: MTT assay. In vivo: Animal model: Wistar rats with experimentally induced HCC (DEN/AAF/CCl4). Assays used: IHC (Ki-67), WB (HGF/c-Met, PI3K/Akt/mTOR), qRT-PCR (Wnt).	In vitro IC50 (µg/mL): HepG2: 190.91 ± 5.17. Huh7: 134.4 ± 4.84. In vivo: Inhibited cancerous lesions growth. Suppressed Ki-67 expression. Downregulated HGF/cMet, Wnt/β-catenin, and PI3K/Akt/mTOR signaling pathways.	[62]
*Silybum marianum*	Methanol extract	Fruits (*Silymarin* component)	In vitro: 0–250 µg/mL. In vivo: 150 mg/kg bw (oral, every other day).	In vitro: Cell lines: Human HCC cell lines (HepG2, Huh7). Assay used: MTT assay. In vivo: Animal model: Wistar rats with experimentally induced HCC. Assays used: IHC (Ki-67), WB (HGF/c-Met, PI3K/Akt/mTOR), qRT-PCR (Wnt).	In vitro IC50 (µg/mL): HepG2: 99.66 ± 3.62. Huh7: 46.97 ± 1.89. In vivo: Inhibited cancerous lesions growth. Suppressed Ki-67 expression. Downregulated HGF/cMet, Wnt/β-catenin, and PI3K/Akt/mTOR signaling pathways.	[62]
*Silybum marianum*	N/A (purchased)	Purchased *Silibinin* flavonoid	In vitro: 0–250 µg/mL. In vivo: 5 mg/kg bw (oral, every other day).	In vitro: Cell lines: Human HCC cell lines (HepG2, Huh7). Assay used: MTT assay. In vivo: Animal model: Wistar rats with experimentally induced HCC. Assays used: IHC (Ki-67), WB (HGF/c-Met, PI3K/Akt/mTOR), qRT-PCR (Wnt).	In vitro IC50 (µg/mL): HepG2: 58.72 ± 2.03. Huh7: 17.18 ± 0.97. Most potent anti-HCC agent tested in HepG2 and Huh7 cells. In vivo: Inhibited cancerous lesions growth. Suppressed Ki-67 expression. Downregulated HGF/cMet, Wnt/β-catenin, and PI3K/Akt/mTOR signaling pathways.	[62]

**Table 6:** Anti-cancer activity of *Carduus pycnocephalus*.

Plant Name	Solvent Used for Extraction	Plant Part	Dose Used	Experimental Model	Results	References
*Carduus pycnocephalus*	Methanol	Crude plant extract	31.3, 62.5, 125, 500, and 1000 µg/mL (five concentrations prepared in serial dilution)	In vitro: Cell line: HePG-2 (hepatocellular carcinoma) human tumor cell line. Assay used: MTT (3-(4,5-dimethylthiazolyl-2)-2,5-diphenyltetrazolium bromide) colorimetric assay.	IC50 = 46.2 µg/mL (indicated a moderate cytotoxic effect)	[66]

**Table 7:** The anti-cancer activity of *Glebionis segetum*.

Plant Name	Solvent Used for Extraction	Plant Part	Dose Used	Experimental Model	Results	References
*Glebionis segetum* L. Fourr.	70% Ethanol extract	Aerial parts	Concentrations ranging from 100 to 0.01 µg/mL (gradual decrease)	In vitro cytotoxicity screening. Cell lines: HT-29 (human colorectal adenocarcinoma) and MCF-7 (human breast adenocarcinoma). Test used: Sulforhodamine B (SRB) colorimetric assay. (Cells exposed for 72 h).	HT-29: IC50: µg/mL ± 17.11. MCF-7: IC50: >100 µg/mL (the extract showed no or very low cytotoxic activity against these cell lines based on NCI standards).	[60]

**Table 8:** Anti-diabetic, anti-enzymatic, and anti-obesity activities of *Silybum marianum* and the anti-diabetic activity of *Glebionis segetum*.

Plant Name	Solvent Used for Extraction	Plant Part	Dose Used	Methods Used	Results	References
*Silybum marianum*	Methanol (SMLM)	Seeds (Whole extract)	IC_50_ concentration determined	Anti-diabetic assay	IC_50_ = 5.2 ± 0.07 μg/mL (superior to standard acarbose IC_50_ = 377.01 ± 1.06 μg/mL)	[69]
*Silybum marianum*	*n*-hexane (SMLH)	Seeds (whole extract)	IC_50_ concentration determined	Anti-diabetic assay	IC_50_ = 18.02 ± 0.24 μg/mL (lowest potential among tested extracts, but superior to acarbose)	[69]
*Silybum marianum*	Ethanol/Water (MEE)	Whole extract/compounds (flavonolignans identified)	IC_50_ concentration determined	-Amylase inhibition	IC_50_ = 26.5 ± 1.3 μg/mL (significantly stronger than acarbose IC50 = 45.8 ± 1.2 μg/mL)	[41,70]
*Silybum marianum*	Aqueous (boiled distilled water)	Seeds (Seed Extract NP4)	Not specified for IC_50_/inhibition %	-Amylase inhibition	38.74 ± 1.09% inhibition (highest activity found)	[71]
*Silybum marianum*	Dilute Acetic Acid	Seeds (Fraction F4)	Not specified for IC_50_/inhibition %	-Amylase inhibition (in vitro, DNSA method)	68.87 ± 2.94% inhibition	[72]
*Silybum marianum*	Ethanol/Water (MEE)	Whole extract/compounds (flavonolignans identified)	IC_50_ concentration determined	-Glucosidase inhibition (in vitro)	IC_50_ = 18.1 ± 1.7 μg/mL (comparable to acarbose IC_50_ = 17.8 ± 1.1 μg/mL)	[41]
*Silybum marianum*	Aqueous (boiled distilled water)	Seeds (Seed Extract NP4)	Not specified for IC_50_/inhibition %	-Glucosidase inhibition (in vitro)	32.62 ± 1.81% inhibition	[72]
*Silybum marianum*	95% Ethanol	Fruits Silychristin A (isolated compound)	IC_50_ concentration determined	-Glucosidase inhibition (in vitro, using *Saccharomyces cerevisiae* enzyme)	IC_50_ = 8.16 ± 1.83 mM	[72]
*Silybum marianum*	EtOAc fraction (from 95% Ethanol extract)	Seeds Compounds 1–16 (isolated)	IC_50_ concentration determined	-Glucosidase inhibition (in vitro, using *Saccharomyces cerevisiae* enzyme)	IC_50_ = 0.38–37.21 μM (Compound 1 was most potent)	[72]
*Silybum marianum*	95% Ethanol	Fruits Silychristin A (isolated compound)	50 mg/kg body weight/day	Anti-diabetic activity (in vivo, oral sucrose tolerance test)	Reduced postprandial hyperglycemia in type 1 diabetic rats	[72]
*Silybum marianum*	95% Ethanol	Fruits Silychristin A (isolated compound)	50 mg/kg body weight/day	Anti-diabetic activity (in vivo, STZ-induced T1DM rats)	Significantly lowered fasting glucose level after 3 weeks; significantly increased serum insulin levels.	[72]
*Silybum marianum*	Dissolved in Physiological saline solution	Derived from fruits/seeds Silymarin/Silibinin (commercial/isolated)	75 mg/kg body weight/day	Anti-diabetic activity (in vivo, STZ-induced diabetic rats)	Produced hypoglycemic effects; significantly decreased glycosylated hemoglobin A1c levels compared to diabetic control.	[72]
*Silybum marianum*	Alcoholic extract	Aerial parts/leaves Silymarin (whole extract)	400 mg/kg (intraperitoneal injection)	Anti-diabetic activity (in vivo, Alloxan-induced diabetic rats)	Caused a major reduction in fasting serum glucose (∼38.42% reduction); showed protective effect against hepatic and renal disorders.	[72]
*Silybum marianum*	Alcoholic extract	Aerial parts/leaves Silymarin (Whole extract)	400 mg/kg (intraperitoneal injection)	Amylase assessment (in vivo parameter)	Reduced amylase concentration by approximately 15%.	[73]
*Silybum marianum*	Isolated compounds from milk thistle seeds	Seeds/Fruits Silibinin/Isosilybin/Silychristin/Silydianin (isolated compounds)	Varies (e.g., 1–50 μM)	PTP1B (Protein Tyrosine Phosphatase 1B) inhibitors (in vitro)	Identified as natural PTP1B inhibitors.	[73]
*Silybum marianum*	EtOAc fraction	Seeds Compound 12 (flavonoid)	IC_50_ concentration determined	PTP1B inhibitory activity (in vitro)	Showed strong PTP1B inhibitory activities compared to oleanolic acid (IC_50_ = 8.35 ± 0.21 μM).	[74]
*Silybum marianum*	N/A (standard drug containing Silymarin)	N/A (derived from seed extract/standard protection) Seed extract/caps/standard drug	100 mg/kg bw (oral administration in rats)	*α*-L-Fucosidase (FS) assay (colorimetric examination in rat serum)	FS activity decreased significantly: 0.57 ± 0.37 U/L (Hepaticum group) compared to 1.48 ± 0.21 U/L (Control group)	[75]
*Silybum marianum*	Aqueous extract (used as reducing/capping agent for CuO-NPs)	Seeds (seed extract)	200 mg mL^−1^ (CuO-NPs)	Alpha-Amylase inhibition assay	35.5 ± 1.54% inhibition	[75]
*Silybum marianum*	Aqueous extract (used as reducing/capping agent for CuO-NPs)	Seeds (seed extract)	200 mg mL^−1^ (CuO-NPs)	Lipase inhibition assay	80.5 ± 0.91% inhibition	[75]
*Silybum marianum*	Aqueous extract (used as reducing/capping agent for CuO-NPs)	Seeds (seed extract)	200 mg mL^−1^ (CuO-NPs)	Urease inhibition assay	78.4 ± 1.26% inhibition	[75]
*Silybum marianum*	N/A (in vitro system)	N/A Silibinin (pure compound)	32.2 μM (IC_50_)	Xanthine Oxidase (XO) inhibition assay (inhibition of uric acid formation)	IC_50_ of 32.2 μM (inhibition of uric acid formation)	[71]
*Silybum marianum*	N/A (in vitro system)	N/A Silibinin (pure compound)	10 μM	NADPH Oxidase inhibition assay (in PMA-stimulated cell lysate)	50% inhibition	[71]
*Silybum marianum*	N/A (in vitro system, human granulocytes)	N/A Silibinin (pure compound)	15 μM (IC_50_)	5-Lipoxygenase (LOX) pathway inhibition (inhibition of LTB_4_ formation)	IC_50_ values of 15 μM for LTB_4_ formation	[71]
*Silybum marianum*	N/A (in vitro system, human granulocytes)	N/A Silibinin (pure compound)	14.5 μM (IC_50_)	5-Lipoxygenase (LOX) pathway inhibition (inhibition of LTC_4_/D_4_/E_4_/F_4_ formation)	IC_50_ values of 14.5 μM for LTC_4_/D_4_/E_4_/F_4_ formation	[71]
*Silybum marianum*	N/A	N/A Silibinin (pure compound)	50 mg/kg/BW (supplementation in rats)	Xenobiotic metabolizing enzymes assessment: Cytochrome b5 reductase activity	Supplementation modulates xenobiotic metabolizing enzymes, including decreasing activity of ROS-producing cytochrome b5 reductase	[71]
*Silybum marianum*	N/A (administered in diet)	Seeds (extract contained 80% Silymarin, 20% soy lecithin) (SME)	200 mg/kg/day	HFD-induced obesity rat model (11 weeks administration). Measured: final body weight (g), final BMI (g/cm), serum leptin level (pg/L), liver weight (g).	Significantly decreased final body weight, final BMI, and liver weight (*p* < 0.05) vs. HFD control. Significantly decreased serum leptin levels (*p* < 0.01) vs. HFD control.	[76]
*Silybum marianum*	N/A (administered in diet)	Seeds (extract contained 80% Silymarin, 20% soy lecithin) (SME)	200 mg/kg/day	HFD-induced obesity rat model (4 weeks therapeutic administration). Measured: serum leptin level (pg/L).	Significantly decreased serum leptin levels (*p* < 0.01) vs. HFD control.	[76]
*Silybum marianum*	80% ethanol	Seeds (SMEE)	150 mg/kg bw/day (oral gavage for 8 weeks)	DIO rat model (high-fat diet). measured: body weight gain (as % of baseline)	Body weight gain was 200.39% of baseline, a significant decrease (*p* ≤ 0.05) compared to model control (221.81% of baseline).	[77]
*Silybum marianum*	80% ethanol	Seeds (SMEE)	300 mg/kg bw/day (oral gavage for 8 weeks)	DIO rat model (high-fat diet). measured: body weight gain (as % of baseline)	Body weight gain was 189.29% of baseline, a significant decrease (*p* ≤ 0.05) compared to model control.	[77]
*Silybum marianum*	80% ethanol	Seeds (SMEE)	450 mg/kg bw/day (oral gavage for 8 weeks)	DIO rat model (high-fat diet). measured: body weight gain (as % of baseline)	Body weight gain was 181.36% of baseline, a significant decrease (*p* ≤ 0.05) compared to model control.	[77]
*Silybum marianum*	80% ethanol	Seeds (SMEE)	600 mg/kg bw/day (oral gavage for 8 weeks)	DIO rat model (high-fat diet). measured: body weight gain (as % of baseline)	Body weight gain was 177.17% of baseline, representing the lowest gain among treatment groups, a significant decrease (*p* ≤ 0.05) compared to model control.	[77]
*Silybum marianum*	Ethyl acetate	Seeds (rich in Silydianin/Silychristin, 51.2%) (SMEE/P)	200 mg/kg/day (3 weeks preventive)	HFD/F-induced metabolic syndrome rat model. Measured: percent weight gain	Percent weight gain (11.1%) was significantly lower (*p* < 0.001) compared to HFD/F control (24.2%).	[74]
*Silybum marianum*	Ethyl acetate	Seeds (rich in Silydianin/Silychristin, 51.2%) (SMEE/T)	200 mg/kg/day (3 weeks therapeutic)	HFD/F-induced metabolic syndrome rat model. Measured: percent weight gain	Significantly decreased weight gain compared to untreated HFD/F control (*p* < 0.05).	[74]
*Silybum marianum*	N/A (pure compound)	N/A (tested in NAFLD rat model) Silibinin	0.5 mg/kg/day	HFD-induced NAFLD rat model. Measured: visceral obesity, visceral fat, lipolysis (adipose triglyceride lipase expression), gluconeogenesis (FoxO1, PEPCK, G6Pase expression).	Prevented visceral obesity and reduced visceral fat. Enhanced lipolysis (up-regulated adipose triglyceride lipase) and inhibited gluconeogenesis (down-regulated FoxO1, PEPCK, G6Pase).	[78]
*Silybum marianum*	N/A (administered in diet)	N/A (tested in HFD mouse model) Silymarin	40 mg/100 g diet	HFD-induced obesity mouse model. Measured: body weight loss and epididymal fat mass.	Resulted in body weight loss and a reduction in epididymal fat mass.	[78]
*Silybum marianum*	Cold press	Seed oil (low Silymarin) Milk thistle oil (MTO)	80 mg kg^−1^ day^−1^ (0.1% oil/50 gm bw/day)	HFD-induced obesity mouse model (8 weeks treatment). Measured: body weight, oxygen consumption (VO_2_), adipose tissue molecular markers (mitochondrial function, IR signaling).	Attenuated HFD-induced obesity and weight gain (*p* < 0.05). Increased oxygen consumption (VO_2_) to control levels. Increased markers of mitochondrial fusion (Mfn1) and browning of white adipose (CREG1, UCP1, FGF21, SIRT1).	[78]
*Silybum marianum*	N/A (in vitro assays)	N/A Silychristin	0.90 µM (K*I*)	Carbonic Anhydrase (hCA) inhibition assay: hCA VII	Highly effective and selective for hCA VII, with KI of 0.90 µM.	[75]
*Silybum marianum*	N/A (in vitro assays)	N/A Isosylibin A	0.92 µM (K*I*)	Carbonic Anhydrase (hCA) inhibition assay: hCA VA	Equipopent inhibitor of hCAs VA and VII. hCA VA inhibition: K*I* of 0.92 µM.	[75]
*Glebionis segetum*	Essential oil (obtained via hydrodistillation)	Aerial parts (dried)	IC_50_ concentration	Alpha-glucosidase inhibitory activity	0.967 ± 0.006 mg/mL	[26]

**Table 9:** The anti-inflammatory activities of *Picnomon acarna*.

Plant Name	Solvent Used for Extraction	Plant Part	Dose Used	Methods Used	Results	References
*Picnomon acarna*	70% ethanol	Full plant	10 g/mL	Inhibition of Tumor necrosis factor-alpha (TNF-α) in human monocytes	100–80% inhibition	[61]
*Picnomon acarna*	Methanol	Full plant	10 g/mL	Inhibition of Tumor necrosis factor-alpha (TNF-α) in human monocytes	80–60% inhibition	[61]

**Table 10:** The anti-inflammatory activity of *Silybum marianum*.

Plant Name	Solvent Used for Extraction	Plant Part	Dose Used	Methods Used	Results	References
*Silybum marianum*	Dissolved in 100% DMSO	Purified Silymarin	36 μg/mL	In vitro: Inhibition of LPS-induced Cyclooxygenase-2 (COX-2) mRNA expression in human fibroblast cells (real-time PCR)	Significantly inhibited COX-2 mRNA expression (*p* < 0.001)	[82]
*Silybum marianum*	Ethanol (EtOH 96%)	Seeds	Equivalent to 1.0 g seeds powder/kg body weight/day (oral)	In vivo: Experimental nonalcoholic steatohepatitis (NASH) in rats (MCD diet); reduction in hepatic TNF- *α* mRNA (Real-time PCR)	47% decrease in elevated TNF-*α* mRNA levels	[77]
*Silybum marianum*	Ethanol (EtOH 96%)	Seeds	Equivalent to 1.0 g seeds powder/kg body weight/day (oral)	In vivo: NASH in rats (MCD diet); reduction in hepatic TGF-*β* mRNA (Real-time PCR)	26% decrease in elevated TGF-*β* mRNA levels	[77]
*Silybum marianum*	Ethanol (EtOH 96%)	Seeds	Equivalent to 1.0 g seeds powder/kg body weight/day (oral)	In vivo: NASH in rats (MCD diet); reduction in JNK phosphorylation (Immunoblot)	Significant decrease in JNK phosphorylation	[77]
*Silybum marianum*	Methanol	Leaf callus	100 mg/kg (oral)	In vivo: Carrageenan-induced rat paw edema model	93.9% inhibition in paw edema	[83]
*Silybum marianum*	Methanol	Leaf callus	100 mg/kg (oral)	In vivo: Formalin-induced rat paw edema model	91.27% inhibition in paw edema	[83]
*Silybum marianum*	Methanol	Leaves	100 mg/kg (oral)	In vivo: Carrageenan-induced rat paw edema model	74.00% inhibition in paw edema	[83]
*Silybum marianum*	Methanol	Leaves	100 mg/kg (oral)	In vivo: Formalin-induced rat paw edema model	85.61% inhibition in paw edema	[83]
*Silybum marianum*	70% aqueous ethanol	Leaves/fruits	50 mg/kg/day (oral)	In vivo: Reduction in serum TNF-α level in DEN/PB-administered nephrotoxic rats	Significantly lowered the elevated serum TNF-α level	[83]
*Silybum marianum*	1% carboxymethylcellulose (CMC) (solution)	Purified silymarin (standard)	10 mg/kg/day (oral)	In vivo: Determination of serum TNF-α level in DEN/PB-administered nephrotoxic rats	Reduced serum TNF-α level from 109.90 ± 5.32 pg/mL (control) to 81.02 ± 3.48 pg/mL	[84]
*Silybum marianum*	99.9% methanol	Callus cultures (Optimized: Continuous Light + 1.0 mg/L Melatonin)	N/A (Extract composition dependent)	In vitro: Enzyme Inhibition (15-LOX)	42.33 ± 1.59% Inhibition	[85]
*Silybum marianum*	99.9% methanol	Callus cultures (Optimized: Continuous Light + 1.0 mg/L Melatonin)	N/A (Extract composition dependent)	In vitro: Enzyme Inhibition (COX-1)	37.15 ± 1.29% Inhibition	[85]
*Silybum marianum*	99.9% methanol	Callus cultures (Optimized: Continuous Light + 1.0 mg/L Melatonin)	N/A (Extract composition dependent)	In vitro: Enzyme Inhibition (sPLA2)	35.70 ± 0.99% Inhibition	[85]
*Silybum marianum*	54.5% aqueous EtOH	Cell suspension cultures (SMCE) (0.5 mg/L Chitosan)	N/A (Extract composition dependent)	In vitro: Enzyme Inhibition (15-LOX)	35.4 ± 1.3% Inhibition	[86]
*Silybum marianum*	54.5% aqueous EtOH	Cell suspension cultures (SMCE) (0.5 mg/L Chitosan)	N/A (Extract composition dependent)	In vitro: Enzyme Inhibition (COX-2)	31.2 ± 1.0% Inhibition	[86]
*Silybum marianum*	Ethanol/Water (Multistep 70%, 50%, H_2_O)	Undifferentiated callus	15–125 μg/mL	In vitro: Suppression of induced IL-6 release in normal human epidermal keratinocytes (HEKn) stimulated by IFN-*γ* (ELISA)	Complete inhibition of IL-6 release in HEKn at concentrations exceeding 60 μg/mL	[87]
*Silybum marianum*	Methanol	Seeds	200–800 μg/mL	In vitro: Inhibition of nitric oxide (NO) production in LPS-stimulated RAW 264.7 macrophages (Griess assay)	Significantly inhibited NO production compared to control (*p* = 0.01 at 200 μg/mL)	[88]
*Silybum marianum*	Methanol	Seeds	2000 mg/kg, gavage	In vivo: Reduction in NO production in LPS-stimulated peritoneal macrophages	Significantly less NO than control (*p* = 0.001)	[88]
*Silybum marianum*	Distilled water (10% *w*/*v* maceration)	Seeds (aqueous extract, AESS)	100 mg/kg (oral, p.o. for 10 days)	In vivo: Acetic acid (AA) 3-induced ulcerative colitis (UC) in rats; reduction in plasma C-reactive protein (C-RP)	Significant drop in C-RP (*p* < 0.05)	[89]
*Silybum marianum*	Distilled water (10% *w*/*v* maceration)	Seeds (aqueous extract, AESS)	1000 mg/kg (oral, p.o. for 10 days)	In vivo: AA 3-induced UC in rats; reduction in plasma TNF-*α* and IL-1*β*	Significant reduction in plasma TNF-α (*p* < 0.05) and IL-1*β* (*p* < 0.05)	[89]
*Silybum marianum*	Dissolved in H_2_O	Seeds (Aqueous Extract, AESS)	IC50 = 25.79 ± 8.74 μg/mL (fMLP-stimulated); 46.12 ± 9.21 µg/mL (PMA-stimulated)	In vitro: Inhibition of reactive oxygen species (ROS) production by human neutrophils stimulated by fMLP (Luminol-amplified chemiluminescence)	Strong inhibition of reactive-oxygen-species (ROS) generation in fMLP- and PMA-activated neutrophils.	[89]
*Silybum marianum*	Dissolved in H_2_O	Seeds (aqueous extract, AESS)	200 μg/mL	In vitro: Inhibition of Neutrophil Gelatinase-Associated Lipocalin (NGAL) release after PMA stimulation (Western blot)	Significantly lowered NGAL release (*p* < 0.001)	[89]

**Table 11:** The anti-inflammatory activity of *Carduus pycnocephalus* and *Glebionis segetum*.

Plant Name	Solvent Used for Extraction	Plant Part	Dose Used	Methods Used	Results	References
*Carduus pycnocephalus* L.	Chloroform	Aerial parts	500 mg/kg I.P.	Formalin-induced rat paw edema model	75.2% Inhibition of edema after 2 h	[25]
*Carduus pycnocephalus* L.	Ethyl acetate	Aerial parts	500 mg/kg I.P.	Formalin-induced rat paw edema model	75.2% Inhibition of edema after 2 h	[25]
*Carduus pycnocephalus* L.	Water (aqueous extract)	Aerial parts	500 mg/kg I.P.	Formalin-induced rat paw edema model	61.9% Inhibition of edema after 2 h	[25]
*Carduus pycnocephalus* L.	Butanol (water saturated)	Aerial parts	500 mg/kg I.P.	Formalin-induced rat paw edema model	59.27% Inhibition of edema after 2 h	[25]
*Carduus pycnocephalus* L.	Acetonitrile	Aerial parts	500 mg/kg I.P.	Formalin-induced rat paw edema model	38% Inhibition of edema after 2 h	[25]
*Carduus pycnocephalus* L.	Ethanol (alcohol extract)	Aerial parts	500 mg/kg I.P.	Formalin-induced rat paw edema model	30% Inhibition of edema after 2 h	[25]
*Carduus pycnocephalus* L.	Petroleum ether	Aerial parts	500 mg/kg I.P.	Formalin-induced rat paw edema model	30% Inhibition of edema after 2 h	[25]
*Glebionis segetum* L. Fourr.	Hydrodistillation (essential oil)	Aerial parts	Tested concentration range: 250–0.49 µg/mL	5-lipoxygenase inhibition activity	IC_50_: 17 ± 3 µg/mL (strong anti-inflammatory activity)	[26]

**Table 12:** Overview of nanoparticles synthesized using *Silybum marianum* extracts and their physicochemical and biological characteristics.

Plant Name	Solvent Used for Extraction	Plant Part	Types of Nanoparticles Synthesized	Applications of Nanoparticles	Results	References
*Silybum marianum*	Water	Seeds	AgNPs (silver)	Anti-bacterial; anti-fungal; anti-oxidant; anti-inflammatory; anti-diabetic; cytotoxicity screening	• Physical characteristics: Spherical AgNPs, size: 13–18 nm, crystalline structure • Biological activities: a. Anti-bacterial and anti-fungal activity: zones of inhibition: 2–13 mm b. Cytotoxic activity: cell viability of HepG2 Liver Cancer: 54.8 ± 0.9% c. Anti-oxidant activity: DPPH assay: 47.9% ABTS assay: 115.9% d. Anti-diabetic activity: α-Glucosidase inhibition: 25.41 ± 1.37% α-Amylase inhibition: 26.78 ± 1.43% e. Anti-inflammatory activity: COX-2 inhibition: 12.52 ± 2.01% COX-1 inhibition: 14.56 ± 0.87%	[96]
*Silybum marianum*	Water	Leaves	AgNPs (silver)	Anti-urogenital disorder; metabolic regulation; anti-oxidant; anti-inflammatory; hormone-modulating (menopausal urinary incontinence)	• Physical characteristics: Spherical AgNPs, Size range: 15–60 nm • Biological activities: a. Hormonal and urogenital activity: ↑ Serum 17β-estradiol, ↑ Urinary bladder weight, ↑ hydroxyproline b. Metabolic and lipid regulation: ↓ weight gain, ↓ Triglycerides, cholesterol, LDL, ↑ HDL c. Hepatoprotective results: ↓ Liver enzymes AST, ALT, and ALP	[100]
*Silybum marianum*	Silymarin	Not reported	AuNPs (gold)	Hepatoprotective; anti-fibrogenic; anti-oxidant; anti-inflammatory	Physical characteristics: Spherical AuNPs, size range: 4–11 nm. • Biological activities: a. Hepatoprotective activity: ↓ ASAT and ALAT b. ↓ Oxidative stress: ↓ MDA levels c. Anti-fibrotic/anti-inflammatory effects: ↓ α-SMA expression, ↓ TGF-β1 levels d. Anti-inflammatory activity: ↓ pNF-kB levels	[101]

## Data Availability

No new data were created or analyzed in this study. Data sharing is not applicable to this article.
